# Heuristic Greedy Scheduling of Electric Vehicles in Vehicle-to-Grid Microgrid Owned Aggregators

**DOI:** 10.3390/s22062408

**Published:** 2022-03-21

**Authors:** Alaa E. Abdel-Hakim, Farag K. Abo-Elyousr

**Affiliations:** 1 Department of Computer Science in Jamoum, Umm Al-Qura University, Makkah 25371, Saudi Arabia; 2 Electrical Engineering Department, Assiut University, Assiut 71516, Egypt; farag@aun.edu.eg

**Keywords:** hybrid microgrids, energy scheduling, heuristic optimization, electric vehicle, greedy algorithm, uncertainty

## Abstract

In on-grid microgrids, electric vehicles (EVs) have to be efficiently scheduled for cost-effective electricity consumption and network operation. The stochastic nature of the involved parameters along with their large number and correlations make such scheduling a challenging task. This paper aims at identifying pertinent innovative solutions for reducing the relevant total costs of the on-grid EVs within hybrid microgrids. To optimally scale the EVs, a heuristic greedy approach is considered. Unlike most existing scheduling methodologies in the literature, the proposed greedy scheduler is model-free, training-free, and yet efficient. The proposed approach considers different factors such as the electricity price, on-grid EVs state of arrival and departure, and the total revenue to meet the load demands. The greedy-based approach behaves satisfactorily in terms of fulfilling its objective for the hybrid microgrid system, which is established of photovoltaic, wind turbine, and a local utility grid. Meanwhile, the on-grid EVs are being utilized as an energy storage exchange location. A real time hardware-in-the-loop experimentation is comprehensively conducted to maximize the earned profit. Through different uncertainty scenarios, the ability of the proposed greedy approach to obtain a global optimal solution is assessed. A data simulator was developed for the purposes of generating evaluation datasets, which captures uncertainties in the behaviors of the system’s parameters. The greedy-based strategy is considered applicable, scalable, and efficient in terms of total operating expenditures. Furthermore, as EVs penetration became more versatile, total expenses decreased significantly. Using simulated data of an effective operational duration of 500 years, the proposed approach succeeded in cutting down the energy consumption costs by about 50–85%, beating existing state-of-the-arts results. The proposed approach is proved to be tolerant to the large amounts of uncertainties that are involved in the system’s operational data.

## 1. Introduction

### 1.1. Motivation

By optimizing the charge/discharge profiles of the vehicle to grid (V2G) at a parking lot, microgrids can effectively handle and monitor V2G while maximizing the economic revenue and the satisfaction of vehicles stakeholders [1,2]. Due to the recent technological advances, governmental incentives, and modern battery development, EVs have been resurged. Furthermore, the EVs development would continue because of the increased sales, proactive strategies, and the support of giant organizations [3]. Further, EVs lend themselves as a zero-emission transportation mean. Yet, the EVs growing market is faced by several constraints due to (i) the randomness of charging and discharging states of the individual cars at the parking locations, (ii) the availability of the charging slots, (iii) manufacturers’ expectations of mileage per charge vary widely and are not often realistic in real-world driving situations, (iv) the tendency of the drivers to participate effectively in the demand response programs, (v) battery wearing problems due to excessive usage in the real-life programs in the on-grid mode, and (vi) the impact of the EVs upon the energy management strategy and the of the microgrids.

On the other hand, microgrids have a number of characteristics that render the V2G deterministic optimization algorithms, which are unsuitable for EVs prioritization or decision making. Amongst such features are: (i) renewable energy sources (RESs), which are inherently unpredictable and intermittent [4,5], are commonly adopted in microgirds [6] (ii) at the parking lot, V2Gs create stochastic scheduling with time-varying traffic, energy costs, charger capacity, and charging restrictions [7], (iii) typical optimization methods are nonlinear, which means obtaining the global minimum and decision making in a multi-energy subsystem is not guaranteed, (iv) complex correlation profiles between the large number of stochastic parameters that directly impact the economic performance of an V2G, (v) the rapid complexity of energy demand, which is exacerbated by the introduction of specific load variations such as V2G and the coronavirus pandemic impacts, mandates the creation of future suggestion and possibility validations for future smart grids, and (vi) coronavirus pandemic influences both the generation and load and thus impacts charging/discharging profiles [8].

Accordingly, we are motivated to propose a novel effective scheduling approach to manipulate all such challenges for maximization of the economic return of hybrid microgrid (HMG) systems by maximizing the revenue or minimizing the operational costs. At the same time, since the proposed approach is model-free, it should be robust against various kinds of uncertainties and variations in the operational circumstances. The proposed algorithm is used to prioritize V2G charging/discharging, ensuring low costs and thereby significantly increasing the revenue.

### 1.2. Related Work

Recently, energy storage systems within microgrids have engaged an indispensable part [9,10]. Yet, on-grid electric vehicles (EVs) and renewables lend themselves as clean energy and flexible deployment energy storage system; however, the integration of electric vehicle to grid (V2G) is confronted by several challenges such as the stochastic nature of V2Gs and renewables, load demands requirements, battery wearing, and market prices.

In the literature, several studies have been conducted to tackle the integration of V2G within microgrids. In [11], a real time charging scheme of the EVs was conducted under different demand response scenarios with remarkable consumptions costs at the parking lots. The authors of [11] concluded that the EV’s prediction models are recommended for future works. Mortaz et al. [12] presented a method to schedule EVs within microgrids with a conclusion that V2G could reduce the total operational costs within microgrids using an optimization model. In [13], θ-modified krill herd was utilized to coordinate microgrids with plug-in EVs. The authors of [13] utilized the stochastic flow using the unscented transform to cover EVs uncertainty and came to a result that total costs could be reduced via manipulating both the generating resources and the V2G. In another research study [14], an EV energy management was investigated via mixed linear programming. The photovoltaic (PV) uncertainty was added to the model. Further, the energy sold or purchased by EVs with stochastic modeling was considered through demand response (DR). The authors of [14] compared between the stochastic and deterministic approach and concluded that there is a need for stochastic modeling for the EV fleet. Koltsaklis et al. [15] introduced a mixed-integer linear programming model to minimize total costs with considering multiple fuel options. The authors set a maximum limit for CO_2_ emissions; however, the energy storage batteries from EVs were not included. In [16], Monte Carlo simulation was utilized to deploy renewables and plugin EVs within microgrids. With the deterministic optimization approach, the authors of [16] concluded that the total operational costs can be mitigated of the microgrid. Zhang et al. [17] introduced an incentive system for EVs based on prioritization and cryptocurrency to reduce the intermittent nature of renewable energy resources. In [18], a coordination of EVs via a deep reinforcement learning (DRL) approach in V2G configuration was described to examine the EV pricing problem. The authors of [18] concluded that smart charging could be holistically analyzed. In [19], linear programming optimization was introduced for ten plugin hybrid electric vehicles (PHEVs) to minimize costs in both grid-connected and islanded modes of operations. In [20], a decision making via DRL-based approach was conducted with an outcome that the energy management could improve the control performance; however, the vehicles were in autonomous working. The integration of modern renewable energy resources and EVs in DR program via EnergyPlan was performed in [21]. The authors of [21] concluded that V2G technology could enhance the coordination between the HMG sectors. The utilization of EV batteries to balance an unbalanced microgrid was introduced in [22]. Mortaz and Valenzuela [23] utilized Bender’s decomposition to calculate the operational costs to find the optimal size and number of the V2G parking lots via Nelder–Mead optimization algorithm with conclusions that V2G had enhanced the long-term electricity cost. The EVs together with renewables for Croatian islands was discussed in [24], in which the V2G was exploited to build a smart energy system. The optimization of EVs with renewable energy resources of a smart energy infrastructure was investigated in [25].

Zdunek et al. [26] developed a scheduling scheme via the binary linear programming. They concluded that the binary linear programming does not enforce the smoothness of the considered scenarios and the scheduling issues of EVs are still open for further future studies. Exploiting the storage capacity of EVs have been discussed by Fathy et al. in [27] via several meta-heuristic algorithms. They demonstrated that competition over resource algorithm had outperformed other algorithms; however, they considered EVs as just a dispatched load. In another work, Hou et al. [28] utilized the multi-objective seeker optimization algorithm to mannage the EV’s insertion within microgrids with conclusions that EVs together with transferable loads has a strong potential to achieve more economic benefits. In [29], Zhang et al. introduced a smart charging management for EV fleet. Similar research studies were conducted in [30,31]. The fire fly algorithm was utilized by Sufyan et al. in [32]. They demonstrated that EVs charging became more beneficial for EV owners with more penetration of renewables.

Several techniques were utilized in the literature to address V2G related issues. Multi-agent approach was utilized by Egbue et al. [33] for sustainable integration of a greater number of V2G. A contract-based utilization of V2G via mixed integer linear programming was introduced in [34], in which a benefit could be achieved for the microgrid and the EVs. Nash bargaining theory was adopted by Sarparandeh et al. [35]. Aiasghari et al. [36] utilized the DR program together with ARIMA model to reproduce the EVs and other introduced by uncertainties scenarios. The Q-learning with Markov chain was introduced by Kim to model a smart building [37]. Liu et al. [38] combined the transactive with model predictive control approach. Motalleb et al. [39] utilized a meta heuristic and complex valued neural networks.

In [40], a notion energy storage merchant was introduced, thereby mitigating the risk of revenue loss. In another research [41], Mortaz et al. introduced an optimization model for placing and sizing the V2G facility means with a conclusion that V2G program could enhance the long-term feasibility economics. An energy scheduling with the priority given to the islanded operation among microgrids was conducted in [42]. An optimization model of a solar-fed microgrid integrated EVs was conducted in [43]. In [44], a stochastic Stackelberg game was conducted whereby EVs were integrated. Nevertheless, EVs were handled as a local load and the user should formulate energy consumption strategy. A predictive-based discrete event was presented by Ferro et al. [45]; however, the EVs were considered as a load.

Recently, machine learning has been utilized in several engineering problems including the considered problem herein, e.g., [46,47,48]; however, the system modeling in machine-learning-based approaches is not an easy task. It requires sophisticated data preparation and collection for training, validation, and testing.

Lu et al. [49] have presented a greedy approach for energy-efficient scheduling during the production process of the EVs rather than their real-time operation management. They utilized a greedy deterministic approach to find the optimal solution of a bi-objective function through Pareto-front technique. The main purpose was to minimize both the total energy consumption in kWh and the make span in minutes of heterogeneous EVs production. The deterministic approach implies that decision variables have minimum and maximum constraints through which the greedy optimizer chooses the optimal values to meet the objective function. The energy scheduling during the production is a bit different from the problem of microgrid operation, which is considered in this work. The problem here is stochastic in nature. It is based on the arrival and departure rates of the EVs, which vary randomly at certain intervals, which makes it more sophisticated.

### 1.3. Contributions of the Paper

As discussed above, some existing approaches optimize economic operations by building exhausting stochastic models, e.g., [12,13,14,15,16,17,18,19,20,21,22,23,24,25]. Others take an easier way of considering entire EVs battery resources as passive loads, e.g., [27,28,29,30,31,32], burning up a valuable energy compensation resource in rush hours. More sophisticated AI approaches use learning for the same purpose [46,47,48]. Generally, decision making to charge/discharge or leave an EV just for parking is rare in the literature. Further, existing approaches depend on optimization models that need accurate system representation and adopting optimization algorithms with well-defined objective functions. Given the facts of the high dimensionality and the complex stochastic characteristics of the different systems’ parameters, such optimization-based approaches are relatively complicated.

In this paper, we propose an approach to overcome these issues. The contributions of this work can be summarized as follows:

(1) Developing greedy-based stochastic optimization approach for cost minimization.
(2) Stochastic real-time experimentation energy scheduling of electric vehicles at the parking lot aggregators.
(3) Developing a comprehensive simulator, which generates different scenarios of the EV’s states representing diverse uncertainty states of operation conditions. As the EV’s states might be off-grid, i.e., passive parking, or on-grid, i.e., to charge or discharge while parking, the data simulator thus allows the greedy scheduler to find an economic solution for the microgrid operation and also consider the driver’s satisfaction. The data simulator was developed in a hardware-in-the-loop manner to assess the performance of the proposed greedy scheduler, which takes into account the following policies to conform with the simulator: operation, time discretization, energy generation and consumption, EV’s charging/discharging, financial regulations, and the evaluation stage.
(4) Economic and reliability analysis of integrating electric vehicles as well as the renewables into microgrids.
  The developed greedy approach achieves the following advantages:
  – Simple, yet effective.
  – Model-free since it does not need to build a complicated stochastic model for the V2G system,
  – It is independent of the stochastic nature of the system parameters. So, we do not have to worry about the interactions between the stochastic parameters.
  – Efficient in terms of computation cost.
  – There is no complex objective function to be optimized via the system’s input parameters.
  – There is no need for tedious training processes.

The introduced data simulator generates comprehensive dataset including all the system’s input parameters. The proposed simulator represents the complex stochastic characteristics of the input parameters. It presents an effective tool, which overcomes the traditional data shortage problem in such systems. This simulator was used to generate data equivalent to a total of 500 operational years of the considered system for evaluation purposes of our proposed greedy method. It helps in proving the independence of the proposed method on the stochastic variations of the systems’ parameters. The proposed simulator is flexible enough to accommodate any changes in the operation circumstances or the used stochastic parameters. It is used herein for evaluation purposes only; however, it can be used to generate simulated data for any future algorithm that needs massive data, e.g., for training purposes.

### 1.4. Paper Organization

The remaining of this paper is organized as follows: following this introduction, the system is described and the problem is formulated. The proposed greedy optimization is addressed in Section 3. The data simulator is presented in Section 4. The experimental setup and results are given in Section 5 and Section 6, respectively. Finally, the conclusions are presented in Section 7.

## 2. System Description

**Figure 1:**
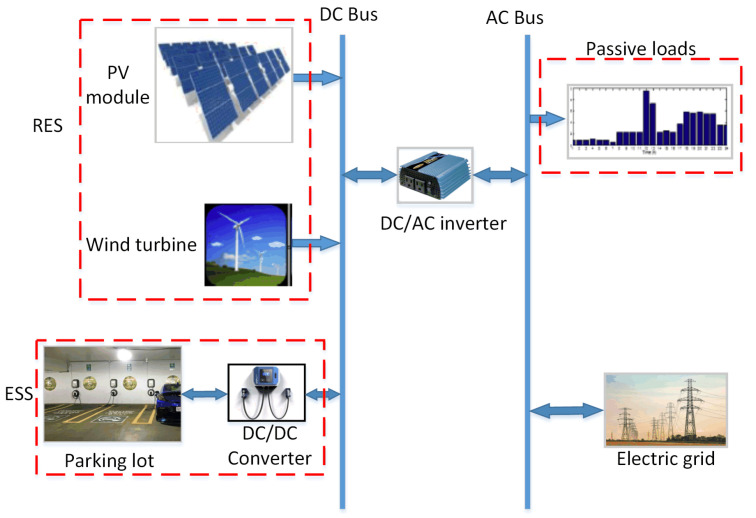
The system under study.

In this work, we tackle the V2G problem from an energy management perspective in a synthetic HMG system. Given a facility with multiple RESs and a V2G-equipped parking lot, as shown in Figure 1, we propose an automated energy operation model. Table 1 summarizes the model parameters that affect the problem sizing. The main objective of this model is to manage the energy consumption in the facility in order for obtaining the optimum economic operation cost. In this section, the components of the system, on which the proposed model is based, are comprehensively explained.

The V2G network facility includes three types of energy sources/loads, as demonstrated in Figure 1. At the DC bus there are the solar photovoltaic (PV) modules, wind turbine (WT), and stochastic energy storage system (ESS) at the parking lot facility. The AC bus incorporates the passive load and the utility grid. These sources and loads represent the major components of the evaluation and testing setup for the proposed framework. These components are sufficiently generic such that it can easily accommodate most of the customization requirements of any given system. In the following subsections, we explain the details of each component on both AC and DC sides.

### 2.1. Passive Loads

A passive time-varying load consists of common loads of any premises, e.g., lighting, air conditioning, etc. The load amounts are stochastically time-dependent and strongly correlated to the premises type and activities. For instances, if it is an administration building including offices, banks, companies, etc., then the peak load times are during the common business hours. If it is a shopping mall with theaters and stores, the peak load times will be during evenings, weekends and holidays, and so on. All passive loads are integrated in a single resultant load (L(t)).

### 2.2. Renewable Energy Source (RES)

In our model, we integrate all the renewable energy modules regardless their types, capacities, or electrical specifications in one large RES. The generated power of this aggregated RES is calculated by summing up all the energy amounts that are generated by the underlying resources. Equation (1) illustrates how the integrated renewable energy is calculated.

(1) $$E_{res}\left(t\right)=\sum_{i=1}^{N_{res}}rc_{i}\left(t\right)$$

where $E_{res}\left(t\right)$ is the total generated RES energy, $N_{res}$ is the total number of units building up the system’s RES, and $rc_{i}\left(t\right)$ is the energy generated by the *i*th resource at a given instant *t*. The generated energies are stochastic and time-dependent. Further, they are related to the types of the installed resources and the nature of the operation environments. For instance, we can easily imagine the daily energy profile that is generated by the a PV panel in a sunny region versus that of a wind turbine in a coastal city.

### 2.3. Energy Storage System (ESS)

A large energy storage (ESS) is represented as an integrated virtual battery that is comprised by the EV batteries, which are parking inside the considered public parking lot at a specific time. Obviously, the parking lot includes a V2G Control. The total capacity is the integration of the capacities of the parking EV’s batteries. The stochastic nature of this storage is a bit sophisticated. Particularly, it can be looked at as a stochastic multidimensional function whose several time-varying independent variables. These variables are listed as follows:

1. The traffic, i.e., entrance and exit rates, of EVs. This is related to the nature of the neighborhood of the facility. For instance, is it in a city center or a suburban? What is the nature of the neighboring business, e.g., banks, markets, playgrounds… etc.?
2. The parking duration of the vehicles.
3. The capacity ratings of the parking EVs.

The total available stored energy can be expressed by the following equation:

(2) $$E_{ess}\left(t\right)=\sum_{i=1}^{n_{ev}\left(t\right)}E_{ev}^{i}\left(t\right)$$

where $E_{ess}\left(t\right)$ is the total stored energy, $n_{ev}\left(t\right)$ is the number of the parking EVs at a given instance *t*, and $E_{ev}^{i}\left(t\right)$ is the instantaneous charge level of the the *i*th EV.

### 2.4. Public Electric Grid

In this work, we treat the public electricity network as an infinite energy source/sink. This is valid under the assumption of 24/7 network availability. The public network’s main functionality, in the proposed model, is to compensate for any energy shortage or excess in the premises, i.e., the system’s energy generation and consumption are always balanced. A decision of purchase or sell energies to the public network at a specific instance is the main action of the proposed approach of greedy scheduling.

### 2.5. Power Converter Model

Because the available energy in RES and ESS is DC, a power converter model is required to convert the harvested energy into AC to match the utility grid and the passive load demands. The converter should handle the maximum energy ($P_{l}^{max}$) required by the load and/or delivered to the grid, as shown in Equation (3).

(3) $$P_{con}\left(t\right)=\frac{P_{l}^{max}}{\eta_{con}}$$

where $\eta_{con}$ is the converter efficiency.

## 3. Greedy Scheduler

The main objective of this study is to determine the V2G charge/discharge profile, which achieves the maximum revenue or the minimum cost in a greedy manner. The main hypothesis is that this greedy local optimization strategy turns to achieve global minimization of the operational cost on the long run. To formulate this greedy local optimization, assume a minimization function *J* as shown in Equation (4). It minimizes the operational costs ($P_{ope}^{c}$), the exchange energy costs between the HMG system and the main grid ($P_{exc}^{c}$), and the total V2G charging/discharging expenses ($P_{V2G}^{c}$).

(4) $$J\left(t\right)=min(P_{ope}^{c}\left(t\right)+P_{exc}^{c}\left(t\right)+P_{V2G}^{c}\left(t\right))$$

(5) $$P_{ope}^{c}\left(t\right)=C_{mpv}P_{pv}\left(t\right)+C_{mWT}P_{WT}\left(t\right)$$

(6) $$P_{exe}^{c}\left(t\right)=C_{buy}\left(t\right)P_{buy}\left(t\right)-C_{sell}\left(t\right)P_{sell}\left(t\right)$$

(7) $$P_{V2G}^{c}\left(t\right)=\left(C_{ch}\left(t\right)P_{ch}-C_{dch}\left(t\right)P_{dch}\right)+\gamma \left(P_{ch}-P_{dch}\right)$$

where *C${}_{mpv}$* and *C${}_{WT}$* are the maintenance costs of the PV and wind turbine in $/kWh, *P${}_{pv}\left(t\right)$* and *P${}_{WT}\left(t\right)$* are the instantaneous aggregated energy production of the PV and wind turbine, respectively. *C${}_{buy}\left(t\right)$* and *C${}_{sell}\left(t\right)$* are the prices of the purchased/sold energy from and to the main grid, respectively, while *P${}_{buy}\left(t\right)$* and *P${}_{sell}\left(t\right)$* are the corresponding aggregated energies in kWh. *C${}_{ch}\left(t\right)$*/*C${}_{dch}\left(t\right)$* are the instantaneous prices of the charge/discharge energy of the V2G batteries and *P${}_{ch}$*/*P${}_{dch}$* are the corresponding aggregated energies in kWh. γ is the battery wearing costs in $/kWh.

These ingredients of the objective function are highly dependent on the system’s electric and financial components, which are discussed later in this section. These components represent stochastic independent time-varying variables of generated/consumed energy amounts. From the proposed model’s perspective, the load and renewable energy resources are considered non-dispatchable because they are out of the model’s operation scope. In other words, $P_{ope}^{c}\left(t\right)$ is uncontrollable by the proposed model. The only controllable component by the proposed model is the energy delivered to or withdrawn from the parking EVs, which is melted down to $C_{ch}$ and $C_{dch}$, respectively. At any time instant *t*, the generated renewable energy $E_{res}\left(t\right)$ and the loads L(t), we need to estimate the optimal amount of energy, which is used to charge or discharge the parking EVs with objective to minimize the total cost of energy purchase or to maximize the total revenue from energy sell, whichever applicable.

The optimization process of the proposed model is restricted by some constraints. Particularly, the energy amounts taken from or delivered to the public network are dependently adjusted to result in a zero resultant balanced energy as:

(8) $$E_{res}\left(t\right)+E_{buy}\left(t\right)-E_{sell}\left(t\right)+y^{-}\left(t\right)-y^{+}\left(t\right)=L\left(t\right)$$

where $y^{-}\left(t\right)$ and $y^{+}\left(t\right)$ are the withdrawn and provided energy from and to the EVs at a time slot *t*, respectively, *E${}_{buy}\left(t\right)$* and *E${}_{sell}\left(t\right)$* are the energies purchased or sold to the microgrid at a time instant *t*. The battery state of charge (SoC) is constrained by the inequality of Equation (9). The values of the upper and lower limits in this inequality are set for better battery health according to the recommendations of most manufacturers.

(9) 0.2≤SoC≤0.9

In addition, the status of the battery on the on-grid mode, is given as:

(10) $$h_{t}^{+}\left(t\right)+h_{t}^{-}\left(t\right)\leq 1,\;\;\forall \;h_{t}^{+}\left(t\right)\;\&\;h_{t}^{-}\left(t\right)\in \left\{0,1\right\}$$

where $h_{t}^{+}\left(t\right)$ is the on-grid sate of an EV battery and $h_{t}^{-}\left(t\right)$ corresponds to the off-grid state. At a charging/discharging spot, the charger spot capacity ($CH^{max}$) is limited by the thermal features of the transmission lines that links the charger to the utility grid. $y^{+}$(*t*) is bounded by:

(11) $$0\leq y^{+}\left(t\right)\leq \sum_{i=1}^{N_{sp}}h_{t}^{+}\left(t\right)\times CH^{max}$$

where, $N_{sp}$ is the number of charging/discharging spots. $y^{-}\left(t\right)$ is governed by:

(12) $$0\leq y^{-}\left(t\right)\leq \sum_{i=1}^{N_{sp}}h_{t}^{-}\left(t\right)\times CH^{max}$$

The stochastic nature of almost every piece of the considered problem makes finding a model that can simultaneously accommodate all of these amounts of uncertainties very challenging. The proposed greedy model avoids all the complications, uncertainties, and correlation of the input parameters by formulating the problem as a local optimization issue in order for estimating immediate maximum gain. Particularly, given the instantaneous values of energy and financial time-dependent parameters, in addition to the parking lot information in terms of $n_{ev},SoC_{i}$, and the parking age of each EV, what would be the best operation decision in terms of charging or discharging EVs, and hence should we sell or purchase energy to or from the public network?

By its nature, the proposed system is continuous in the time domain; however, for modeling simplification, we opted to discretize the independent time variables into a number of fixed-length intervals or time slots, $n_{t}$. For example, if a week-length period is considered for modeling with 15-min fixed-length discrete intervals, then $n_{t}=\frac{60\;\times \;24\;\times \;7}{15}$. $n_{t}$ is a configurable parameter that determines the desired status changes frequency and the operation pattern periodicity. The status change refers to the average frequency in which the elements of the input parameter change, e.g., the traffic of vehicle entrance and exit. The operation pattern periodicity indicates the state repetition depending on rush hours or high seasons.

The instantaneous system status is described by an aggregation of status vectors, $s_{i}$, as shown in Equation (13).

(13) $$S=\{s_{i}:s_{i}\in R^{7N_{sp}+7};\forall i\in \left\{1,\ldots ,n_{t}\right\}\}$$

where $N_{sp}$ is the capacity of the parking lot. The set, S, describes the entire system status over the considered modeling period. S comprehensively describes all the details of the system at a specific time slot *i*. Each vector $s_{i}$ comprises a total number of $7N_{sp}+7$ elements: seven scalars representing the instantaneous values of L(t), the total $E_{res}$, and the instantaneous sell and purchase energy prices to and from the public network, as well as sell and purchase to and from the parking EVs. These seven scalars are concatenated with a number of $N_{sp}$7D-spot vectors. Each of these spot vectors represents the instantaneous status of a parking spot. The parking spot status is a collection of seven values, which are: the binary occupancy status, the parking age of the vehicle measured in number of time slots, the current $SoC_{i}$, the EV gained charge since entered to the parking lot, a Boolean charging status flag, and another discharging flag.

The input of the model is a vector, $I=T\left(s_{i}\right)\in R^{10}$ that is generated using a linear aggregation transform $T:R^{7N_{sp}+7}\to R^{10}$, as shown in Equation (14).

(14) $$T\left(s_{i}\right)=\left[\begin{array}{cc} ID & \\ N_{ch} & \\ N_{dch} & \\ N_{ch\_dch} & \\ E_{res} & \\ L(t) & \\ Buy\_net & \\ Sell\_net & \\ Buy\_EV & \\ Sell\_EV \end{array}\right]$$

ID is a time slot index, which represents implicitly the temporal vector profiles. In other words, it implies the traffic and usage patterns of the considered system. For example, for an administrative premises, the traffic nature tends to be weekly periodic. In other words, the facility usage pattern is nearly repeatable every week, i.e., it depends on the weekday and time with no effect of the month. In this case, the week is slotted into a number of time slots every ten or fifteen minutes depending on the average frequency of state changes. In contrary to a hotel facility, for example, the period may be extended to span over the entire 52 weeks of the year in order to better reflection of high seasons.

As per the main hypothesis of this work, the proposed greedy optimization is tolerant to such stochastic variations. Specifically, the taken decisions of the greedy optimizer vary instantaneously to mask out the stochastic variations that may affect the final gain; however, we include the time index parameter in the parameter input vector for investigation and evaluation purposes, not for the core operation of the proposed optimizer.

To formulate the output vector of the proposed optimizer, the actions that can be applied to a V2G power terminal should be identified. A power terminal connected to a parking EV can take one out of three exclusive actions: charge, discharge, or disconnect. The final charging decision is a conclusive decision that summarizes the number of vehicles to be charged, the number of vehicles to be discharged, and consequently the number of disconnected terminals. To determine the proper decision of these numbers, the instantaneous upper bounds of chargeable $\left(N_{ch}\right)$, dischargeable $\left(N_{dch}\right)$, or chargeable–dischargeable $\left(N_{ch\_dch}\right)$ terminals are to be calculated under the constraint of Equation (9), as shown in Equations (15)–(17).

(15) $$N_{ch}=\left|\left\{sp_{i}:SOC\left(sp_{i}\right)+CH^{max}/Q_{n}^{i}\leq 0.9\right\}\right|$$

(16) $$N_{dch}=\left|\left\{sp_{i}:SOC\left(sp_{i}\right)-CH^{max}/Q_{n}^{i}\geq 0.2\right\}\right|$$

(17) $$N_{ch\_dch}=\left|\left\{sp_{i}:CH^{max}/Q_{n}^{i}+0.2\leq SOC\left(sp_{i}\right)\leq 0.9-CH^{max}/Q_{n}^{i}\right\}\right|$$

where $Q_{n}^{i}$ is the *i*th battery capacity and |.| is the set cardinality.

While Equations (15) and (16) calculate the maximum numbers of chargeable and dischargeable vehicles, respectively, Equations (18) and (19) determines their lower bounds in case of all the chargeable/dischargeable spots are assigned to the opposite state.

(18) $$N_{ch\_min}=N_{ch}-N_{ch\_dch}$$

(19) $$N_{dch\_min}=N_{dch}-N_{ch\_dch}$$

The number of the spots to be charged or discharged is determined as one of three possible cases: maximum, minimum, or zero. So, at every time slot, the proposed optimizer greedily decides one out of five possible composite decisions, which are shown in Table 2.

The number of disconnected terminals $\left(d_{nc}\right)$ is dependently determined, once the decided numbers of charging $\left(d_{ch}\right)$ and discharging terminals $\left(d_{dch}\right)$ are decided by the model as in Equation (20).

(20) $$d_{nc}=N_{sp}-\left(d_{ch}+d_{dch}\right)$$

It is clear from Equation (20) that $d_{nc}$ is dependent on $d_{ch}$ and $d_{dch}$. Hence, it can be calculated directly from $d_{ch}$ and $d_{dch}$; therefore, the proposed output vector includes only $d_{ch}$ and $d_{dch}$ because $d_{nc}$ is redundant in this case.

The model evaluates each of the candidate charging decisions, which are listed in Table 2. The candidate action whose instantaneous minimum cost or maximum revenue for the current time slot is selected as the output greedy decision, as shown in Equation (21). Algorithm 1 shows the detailed steps of the greedy scheduling process.

(21) $$GD\left(t\right)=\{Sk|f\left(k\right)=\min_{k}J_{k}\left(t\right),\forall k=1:5\}$$

**Algorithm 1:** The greedy scheduler algorithm.
1:**procedure**Greedy-Scheduler(i,GD)                                          ▹*i* is the timeslot ID2:    $st\leftarrow s_{i}\in S$         ▹ The current state of the system according to Equation (13).3:    I←T(st)                                                             ▹ The input vector, Equation (14).4:    $cost_{min}\leftarrow 10^{8}$5:    **for** k←1 to 5 **do**6:          $cost_{k}\leftarrow J_{k}\left(t\right)$                      ▹$J_{k}\left(t\right)$ is the minimization function evaluated for setting *k* of Table 2.7:          **if** $cost_{k}<cost_{min}$ **then**8:               $cost_{min}\leftarrow J_{k}$9:               GD=Sk(I)    ▹Sk takes the corresponding value to *k*, as shown in Table 2.10:        **end if**11:    **end for**      **return** GD12:
**end procedure**



The decided setting GD(t) does not specify which vehicles are to be charged, which to be discharged, and which terminals are to be disconnected. We follow a charging/discharging assignment policy that favors the EV owner satisfaction by trying to minimize the withdrawn charge from the EV batteries, whenever applicable.

## 4. System Simulator

**Figure 2:**
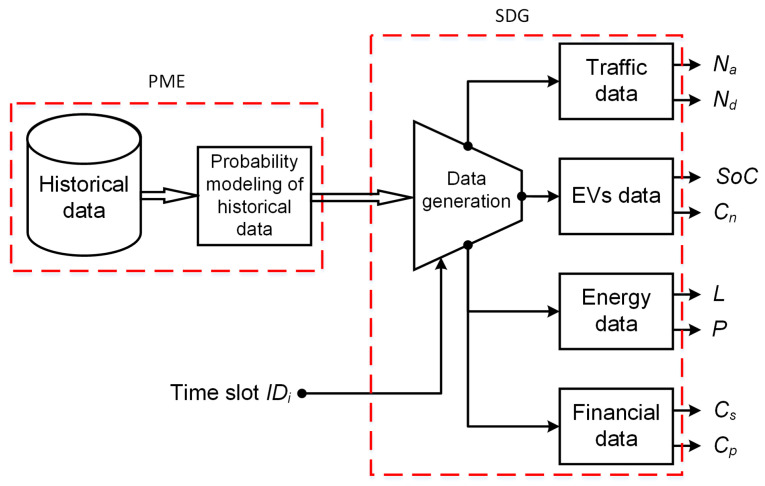
The components of the data simulator.

The difficulty of obtaining a large dataset for the scheduling systems for evaluation purposes mandates generating simulation data that is as realistic as possible. To preserve the big picture of the objective of the intended simulator, we need to keep in mind the real-time daily operation of the considered system. This comprises modeling of four different kinds of data, as shown in Figure 2. The proposed simulator consists of two main components: probabilistic model estimator (PME), and the simulation data generator (SDG). In the following subsections, we discuss the details of operation of these two components.

### 4.1. Probabilistic Model Estimator (PME)

PME is responsible of estimating probabilistic models as accurate as possible. The accuracy of the estimated models depends on how realistic the fed raw historical data is. Due to the natural expected lack of real data, we depend on some historical raw, yet relevant, data for the models estimation. As shown in Figure 2, there are four kinds of data to be generated by the simulator. For each kind, we assume a specific distribution. In the following, we discuss how the models are estimated for each kind.

#### 4.1.1. Traffic Data Model

The traffic data model comprises estimations of two main components, as follows:

- *Parking EVs*:

The number of parking EVs during a time slot is implicitly simulated through a death-and-birth process, as detailed in Section 4.2.1. The main raw data ingredients of that birth-and-birth process are the birth and death, or arrival and departure rates, respectively. We use historical data for arrival and departure rates, as shown in Table 3 [12].

**Figure 3:**
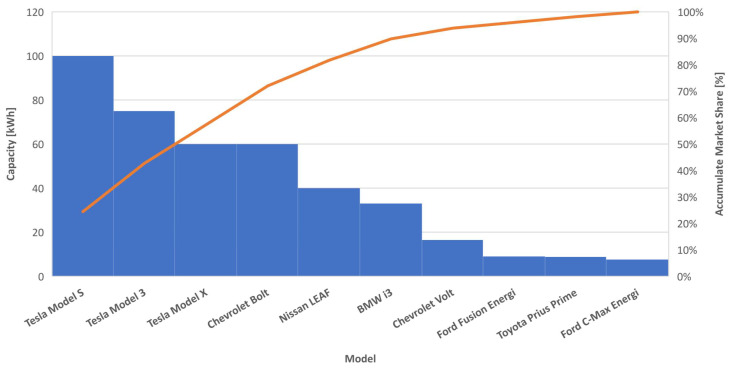
The top best selling cars and their rated capacities.

- *EVs’ rated capacities*:

To model the rated capacities of the parking EVs, we consider the top ten selling vehicles in the US market by the end of 2019 [50]. The model of the parking EVs, hence their capacities, are assumed to be from a weighted uniform distribution relative to the market share of each EV model. The distribution weights are assigned according to the accumulate market share, which is shown in Figure 3.

#### 4.1.2. Energy Data Model

**Figure 4:**
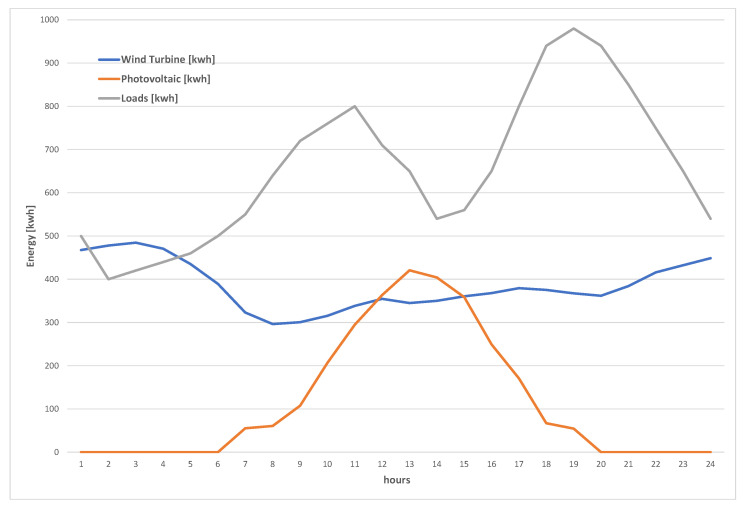
Mean $E_{res}\left(t\right)$ and L(t) daily profiles.

The energy data in the proposed simulator include three main types: (a) the generated wind turbine power, (b) the generated photovoltaic power, and (c) the total consumed power by the facility loads. For the three types of data, we adopted average values of a typical medium-size premises [51,52,53]. Figure 4 shows the distribution of these average values over 24 h. These values are used as the mean values that the data generator uses to generate energy data, as detailed in the Section 4.2.

#### 4.1.3. Financial Data Model

**Figure 5:**
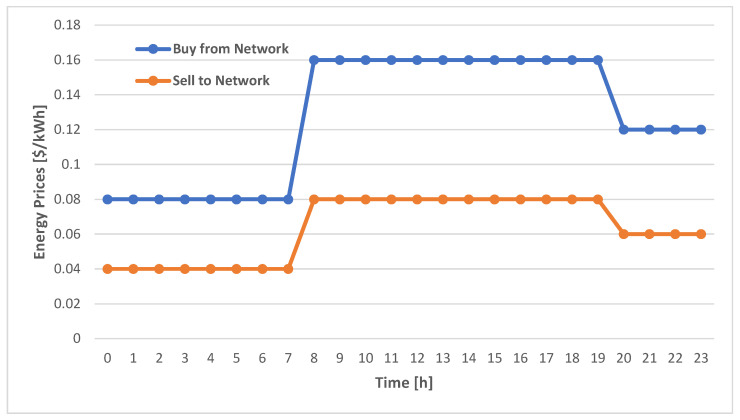
The used raw historical energy prices.

The financial data comprise the prices of the energy trade with the public electric grid. These prices are stochastic and time-dependent. We use historical average energy prices as the raw historical data for the estimated probabilistic model [51,52,53], as shown in Figure 5. Similar to the operation of the energy data model, we consider these values as the pivot values, which represent the means as a function of time. The time-dependent means are used by Monte Carlo simulation to generate the corresponding time-dependent prices, as detailed in a later section.

### 4.2. Simulation Data Generator (SDG)

The core of the proposed simulator contains a data generator, which exploits the probabilistic models generated by the PME, in order to generate the evaluation data. The external input parameter to the SDG is just the temporal parameter represented by a time slot, which encodes the only independent variable of the system, i.e., the weekday and time. SDG includes four independent modules, which generate all the stochastic parameters that build up the input space. Any generated negative random variables are truncated whenever negative values are not applicable. These modules are: traffic, EV, energy, and financial modules.

The stochastic nature of the SDG outputs are diverse. This means that every output set requires different probabilistic model. In the following, we explain the adopted probabilistic modeling technique for each of these modules.

#### 4.2.1. Traffic Module

The arrival/departure process is inhomogeneous. Specifically, the number of the arriving and departing EVs is time-dependent; at the same time it is controlled by time-dependent arrival and departure rates. Moreover, the arrivals and departures are continuous-time variables; therefore, the most adequate model for this process is the inhomogeneous continuous-time Markov chain (ICTMC) [54]. Monte Carlo (MC) simulation is used to realize different datasets and state transitions of the ICTMC model.

Arrivals and departures can be effectively modeled as a birth-and-death process [12]. We assume the arrival of an EV as a birth event, while its departure as a death event. Consequently, the number of the parking EVs at any instant *t* is the process’s state. Since the arrival and departure rates are time-dependent, assume that arrival and departure rates equal λ(t) and $n_{ev}\left(t\right)\mu \left(t\right)$, respectively.

In birth-and-death processes, the holding time is defined as the time elapsed before any change occurs in the process’s state, i.e., before any new birth (arrival) or death (departure) occurrence. The holding time itself is a stochastic variable. According to MC simulation, the holding time can be sampled from its cumulative distribution function (CDF).

#### 4.2.2. EV Module

The EV module generates two values for each of the arriving vehicles. The first one is the capacity of the EV battery ($C_{n}$) in Ah or the corresponding capacity ($Q_{n}$) in kWh. Thus, $C_{n}$ is determined by a random selection of an EV model and make. Further, $C_{n}$ determines the *C*-rate at which an EV takes to fully charge or discharge. This time varies between 10 min for ultra fast charging [55] to several hours at lower charging/discharging rates.

This process is performed through generating a random variable under a finite discrete distribution whose probability mass function is expressed according to the capacitance CDF function, as shown in Figure 3. This generation is performed using the efficient stochastic acceptance algorithm [56]. Generating SoC is much simpler as shown in the following equations, in which $P_{s}$ is the charging or discharging power and $t_{h}$ is the corresponding time.

(22) $$Q_{s}\left(t\right)=Q_{0}\pm \int_{0}^{t_{h}}P_{s}\;dt\;$$

where $Q_{s}\left(t\right)$ is the remaining capacity in kWh, and $Q_{0}$, which is random by nature, are the initial capacity in kWh, respectively. For constant charging/discharging power, Equation (22) can be rewritten as shown in Equation (23), in which the positive sign is used for charging the battery or selling power to an EV and the negative sign refers to discharging or purchasing power from it. As a result, Equations (22) and (23) yield the remaining capacity in kWh denoted as Qs and the battery SoC is assessed as in Equation (24).

(23) $$Q_{s}=Q_{0}\pm t_{h}\times P_{s}$$

(24) $$SoC=\frac{Q_{s}}{Q_{n}}$$

SoC∈[0.2,0.9], as constrained by the inequality of Equation (9), is a uniform random variable. $Q_{n}$ is the battery rated capacity. For the *i*th car, the remaining capacity is consequently determined by Equation (25), from which the the remaining to charge/discharge is evaluated as in Equation (23).

(25) $$Q_{s}^{i}\left(t\right)=SoC_{i}\left(t\right)\cdot Q_{n}^{i}$$

#### 4.2.3. Energy Module

Depending on the fed time slot, ID, to the data generator, the energy module generates the amounts of the requested energy by the instantaneous active loads and the total $E_{res}$. Both values are generated using MC simulation by sampling a normal distribution according to the mean values that have been identified from the historical data, as shown in Section 4.1.2. We use a separate time-varying normal distribution for each hour in the day. Each distribution has a mean value equals the corresponding value of the historical raw data with a specific Z-score. This Z-score guarantees that certain percentage of the generated values by SDG lie within ± half of the mean value. In evaluation experiments, we set the used Z-score to various values in order to investigate the effect of diverse uncertainty levels on the overall performance.

#### 4.2.4. Financial Data Model

The financial data are generated by the financial data module in a similar way to that of the energy module. For a specific run, a global Z-score is used for all relevant modules including the time-dependent energy selling and purchase prices.

## 5. Experimental Design

In the experimentation designed to evaluate the performance of the proposed approach, we follow an end-to-end evaluation strategy. Particularity, we run the system comprehensively and evaluate the overall performance in terms of the final objective, which is minimizing the running cost or maximizing the earned profit. An operation policy is defined for the system to comply with by the simulator’s generated data. Additionally, a set of parameter settings are specified for the simulator, according which the evaluation data sets are generated.

### 5.1. Operation Policy

The operation policy is mainly concerned by determining the technical and financial regulations, which govern the system’s daily processes. Although we follow a specific operation policy in this work, the proposed model is a generic framework, which can fit most custom financial policies that a facility opts to adopt. The operation policy is based on four main facets, which are discussed in the following subsections.

#### 5.1.1. Time Discretization

In reality, the operation domain of the model environment is within a continuous time domain. In other words, all variations in the system are unrestricted by specific time intervals. Particularly, the operation mode is CTMC rather than DTMC; however, working in DTMC mode would not have negative impact on the model operation nor on the evaluation results; therefore, for operation simplicity reasons, we opted to enforce any state variation to occur in a discrete manner at the beginnings of a predetermined tune-able parameter representing the time intervals, Δt.

#### 5.1.2. Energy Generation and Consumption

At all times, regardless the actual loads, the generated renewable energy, $\left(E_{res}\right)$, should be completely consumed. This energy can either be consumed by local loads in the premises, charge EVs, or sold to the public network. In other words, the maximum possible amount of $E_{res}$ is accepted with no capacity restrictions. This is a legitimate assumption, since a major common visionary objective of such facilities is to maximize the generated clean energy, specially when energy storage is available. Further, the public electric grid is considered as an infinite energy source and sink. In other words, energy purchase from or selling to the public network is unlimited and available 24/7, regardless the cost effectiveness. These two assumptions simply mean that the energy generation and consumption have no limits or constraints, thanks to the unrestricted public electric grid supplies.

#### 5.1.3. EVs Charging/Discharging

Aside from energy cost optimization, customer satisfaction is an objective of the proposed model. In terms of charging and discharging processes, reaching complete customer satisfaction conflicts with the primary goal of energy optimization. Nonetheless, the model’s operation policy is adjusted towards achieving the maximum amount of customer satisfaction as possible. From a customer perspective, getting their EV with maximum charging level is the optimum case, whereas picking it with a charge level less than what it was left with makes them dissatisfied; therefore, the operation policy sorts its priorities by putting minimizing discharging from EVs batteries in the highest priority level, i.e., the top priority is to minimize customer dissatisfaction. Then, charging priorities goes up towards increasing the charging levels, i.e., maximizing his/her satisfaction. In the light of this prioritization, the operation policy works in a greedy manner.

To implement this prioritization policy, a sorted list of parking vehicles’ amounts of consumed/earned energy is continuously updated. The top of this sorted list is the vehicle whose largest amount of energy that was discharged by the system. Then the list goes down till the vehicle, which has obtained the maximum charge amount comes at the very bottom of the list. When a specific number of vehicles are decided to be charged, they are picked from the top of the list. The vehicles which are decided to be discharged are picked from the bottom of the list. This kind of prioritization guarantees to keep the amount of vehicles discharged as minimum as possible all the time. This achieves the strategic hypothesis of the proposed greedy scheduler to avoid any stochastic modeling for predicting the departure time of every single EV. So, the minimum customer dissatisfaction is achieved with the uncontrollable exit of the parking vehicles without the need for complex stochastic prediction processes. Additionally, as discussed earlier, $SOC_{i}$ is constrained by Equation (9) for the health of the batteries as recommended by most manufacturer.

#### 5.1.4. Financial Regulations

In the proposed model, we assume stochastic time-variant public network’s energy sell and purchases price. Usually, these prices are country-dependent and determined by energy stock market on short and long term contracts [12,57]. In real-time operation, these prices are fed to the system continuously. On the other side, we assume fixed sell and purchase prices to the EV owners. To keep life easier and to maintain operation simplicity, the EV owners are assumed to consent to their vehicle energy level variation in the parking lot within a charge level of 20–90% of the rated capacity.

### 5.2. Evaluation Data

The proposed system exploits a diverse set of data in terms of energy generation, consumption, prices, and parking lot traffic. All of these data are stochastic and time dependent. For evaluation purposes, researchers in the literature usually use data simulators, e.g., [12]. Depending on precise statistical models for simulation guarantees generation of accurate simulation data that can reliably approximate real data. This is what we achieve using the proposed PME and SDG, which are both explained in Section 4.

## 6. Experimental Results

The main objective of the evaluation procedures, which we follow for the proposed system, is to prove the effectiveness of the proposed greedy scheduler in operational cost saving. So, the most direct metric to evaluate the effectiveness of the proposed scheduler is the integral cost–savings achieved over evaluation periods, which is the ultimate goal of the proposed scheduler. The cost saving metric is measured as the ratio between the running cost before and after applying the proposed method. This is emphasized in the results shown in this section. Additionally, the robustness of the proposed approach against the large and various underlying amounts of uncertainties of the system parameters is emphasized in the conducted experiments. To achieve these goals, we conducted a total of 500 completely independent simulated annual runs of the entire proposed system. We adopted five different global uncertainty levels to govern the stochastic variation of the system parameters. Specifically, we use five different global Z-scores for normally distributed parameters, which are detailed in Section 4. These values are shown in Table 4. The third column of the table shows the portion of the drawn samples that swap along ± half of the mean values of the considered parameter. These five global simulator settings guarantee that the simulator exhibits diverse levels of uncertainties for all the stochastic parameters of the system. As shown in Table 4, while $U_{10}$ implies the largest uncertainty level, $U_{95}$ implies the most deterministic degree of the stochastic parameters. For every uncertainty level, 100 independent runs are executed, which comprises a total of an effective simulation duration of five hundred years. The long effective simulated operational duration guarantees accurate and reliable statistical evaluation results.

**Figure 6:**
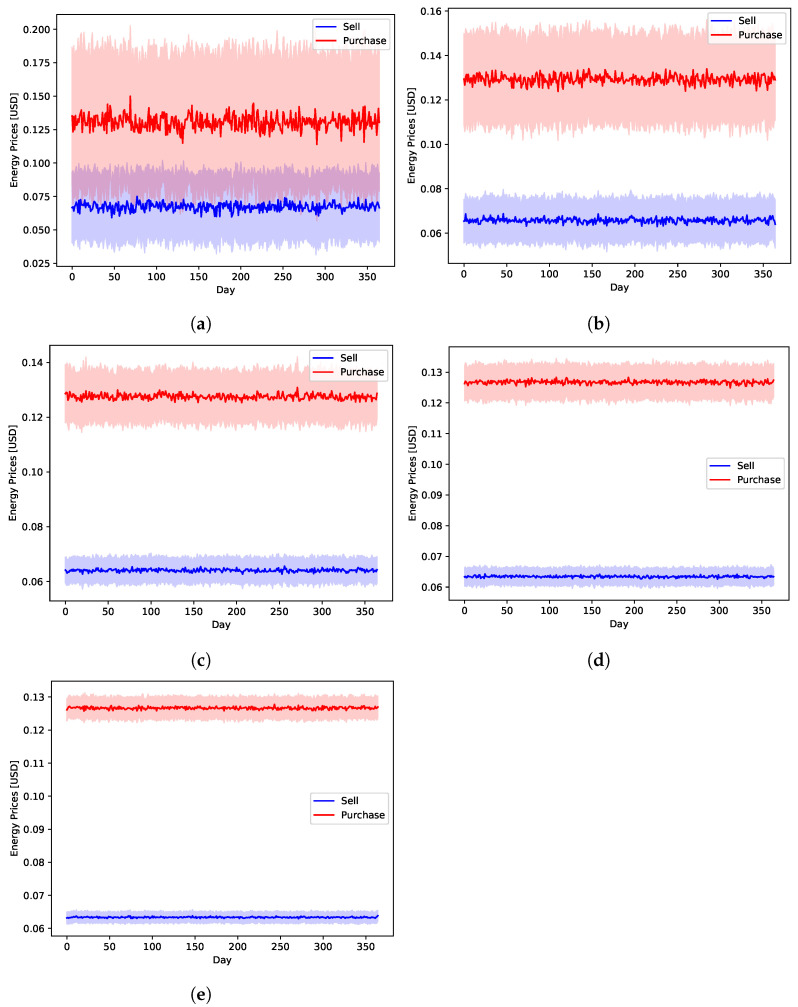
The simulated energy sell and purchase prices to and from the public network with different global uncertainty levels. (**a**) $U_{10}$, (**b**) $U_{25}$, (**c**) $U_{50}$, (**d**) $U_{75}$, (**e**) $U_{95}$.

**Figure 7:**
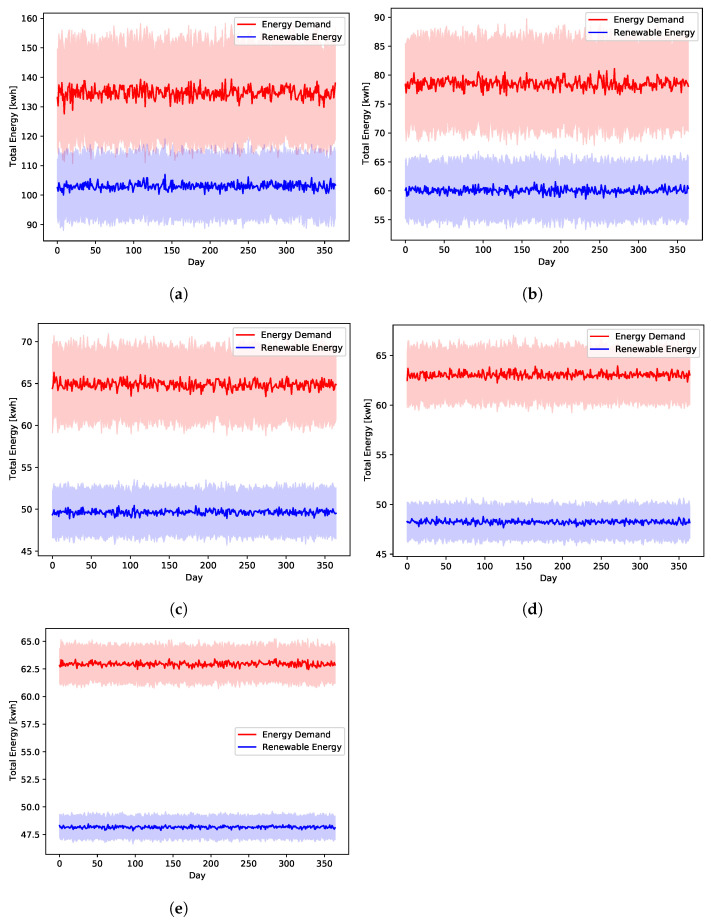
The simulated $E_{res}$ and L(t) with different global uncertainty levels. (**a**) $U_{10}$, (**b**) $U_{25}$, (**c**) $U_{50}$, (**d**) $U_{75}$, (**e**) $U_{95}$.

The main independent stochastic variables of the systems, which are affected directly by the global uncertainty levels, are those produced by the traffic, energy, and financial data modules of Figure 2. This is because the data generator models them to follow normal distributions. The EV data are assumed to be uniformly distributed. So, they are not affected by the variation of the global uncertainty level. Figure 6 shows the average daily energy prices of the public network as generated by the data generator. Similarly, Figure 7 shows the daily average energy generation and demands by the renewable resources and loads, respectively. These two figures highlight the impact of the adopted uncertainty level on the generated values. This is a crucial challenge for any model-based approach, since it has to capture such variations as well as the complex inter-parameter correlations.

**Figure 8:**
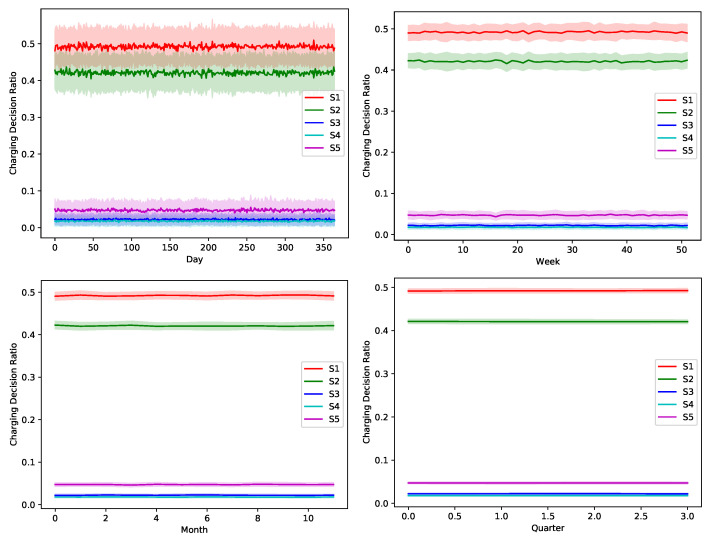
The temporal variation of the adopted charging decision by the greedy algorithm with a global certainty level $U_{10}$.

**Figure 9:**
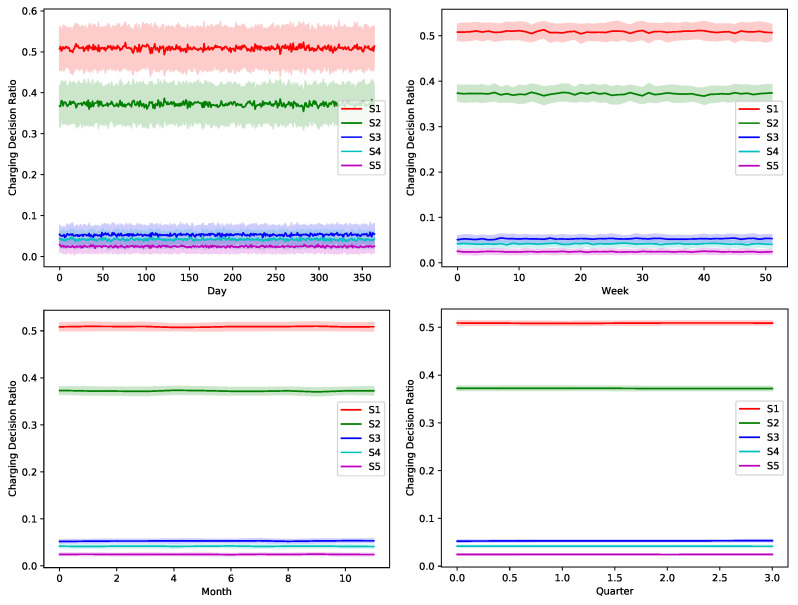
The temporal variation of the adopted charging decision by the greedy algorithm with a global certainty level $U_{25}$.

**Figure 10:**
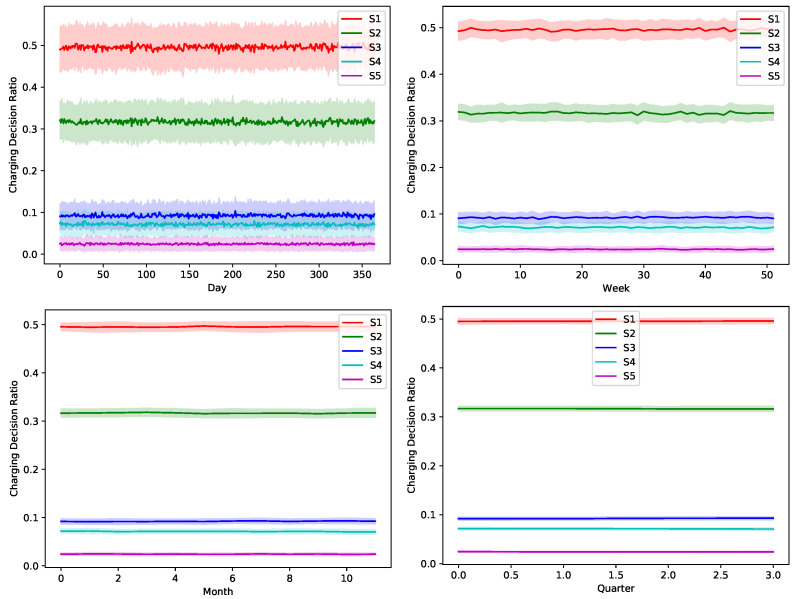
The temporal variation of the adopted charging decision by the greedy algorithm with a global certainty level $U_{50}$.

**Figure 11:**
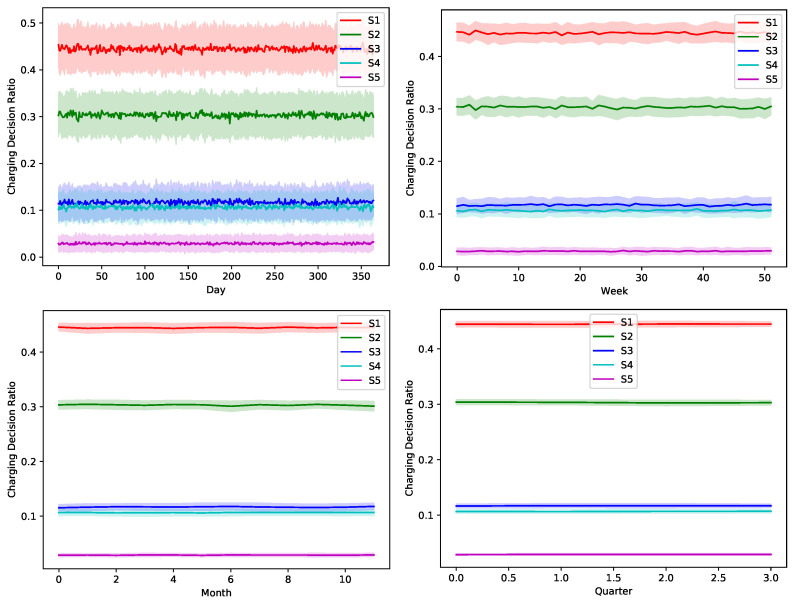
The temporal variation of the adopted charging decision by the greedy algorithm with a global certainty level $U_{75}$.

**Figure 12:**
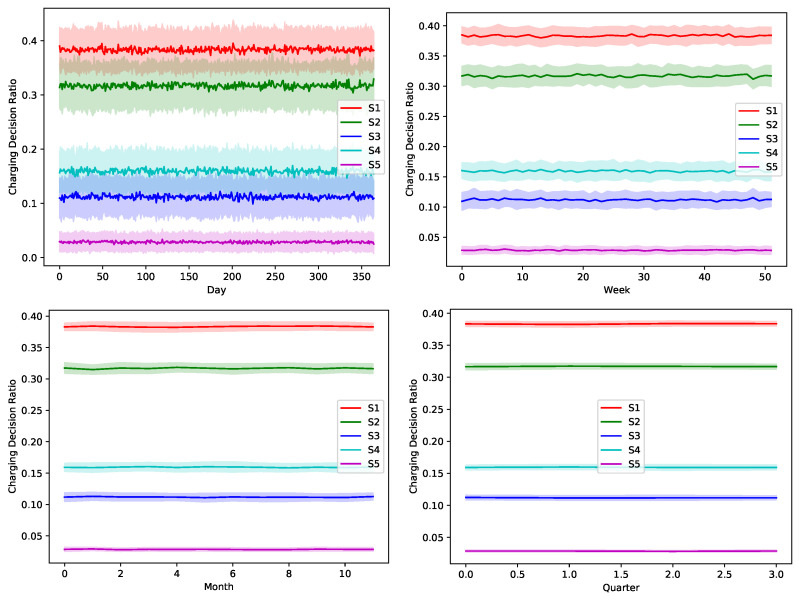
The temporal variation of the adopted charging decision by the greedy algorithm with a global certainty level $U_{95}$.

The proposed greedy algorithm makes instantaneous decision to alternate between different charging/discharging decisions. As shown in Figure 8, Figure 9, Figure 10, Figure 11 and Figure 12, no single scheduler is excluded from the global optimal scheduling process. Although, S3 and S4 usually have a small representation share in the greedy decision, they still exist. This fact emphasizes our argument that the complex stochastic nature of the system makes reaching an optimal scheduling strategy needs very sophisticated stochastic models to capture its complex variations. The figures show how the proposed scheduler adapts with the instantaneous variations in different operation circumstances. Specifically, when we look at these statistics on a daily basis, we can see how the shares largely oscillate around their mean values, while it is relatively stable when the evaluation duration is extended. This is because the shorter the evaluation duration is, the more dynamic the system’s stochastic parameters are. While the mean values of the greedy decisions are affected by the global uncertainty levels, the overall profile in terms of the order of adopted schedulers is almost the same.

Table 5 shows a conclusive statistical illustration of the taken scheduling decisions by the proposed scheduler. The aforementioned reached finding of nonexistence of a unified model-free scheduling policy that can be optimally adopted all the time, is proved herein.

Figure 13, Figure 14, Figure 15, Figure 16 and Figure 17 show the time variation of EVs charging and discharging at the V2G terminals due to the decision taken by the greedy scheduler. The same argument and conclusions that are discussed for Figure 8, Figure 9, Figure 10, Figure 11 and Figure 12 apply herein for the EV terminals charging/discharging.

**Figure 18:**
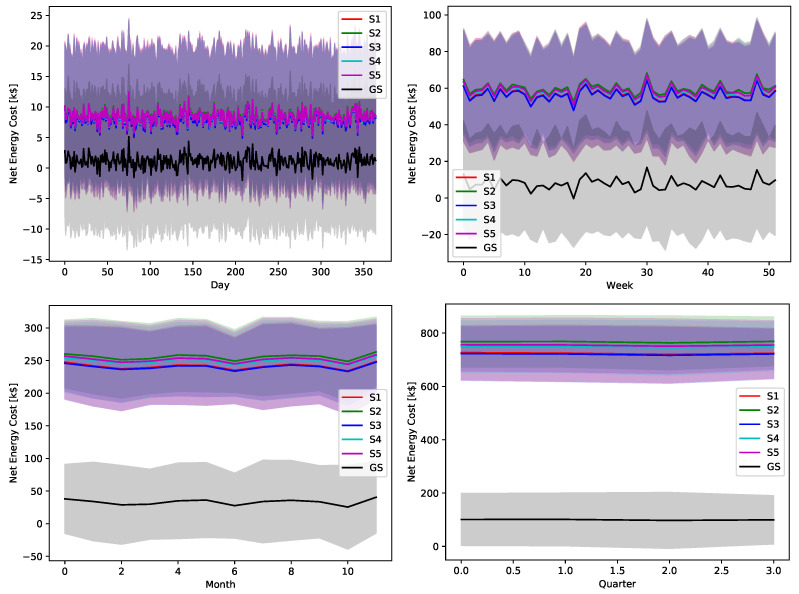
The total operation cost in terms of net energy purchase and sell revenue with a global certainty level $U_{10}$.

**Figure 19:**
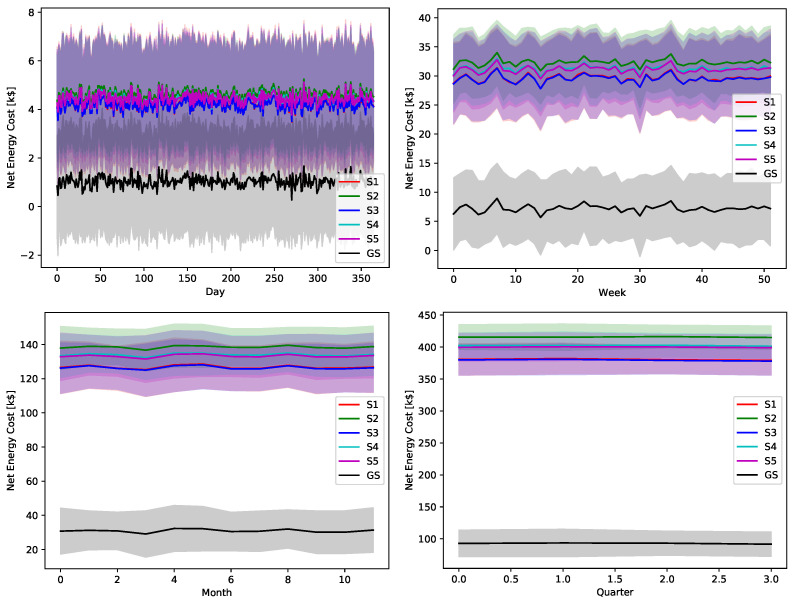
The total operation cost in terms of net energy purchase and sell revenue with a global certainty level $U_{25}$.

**Figure 20:**
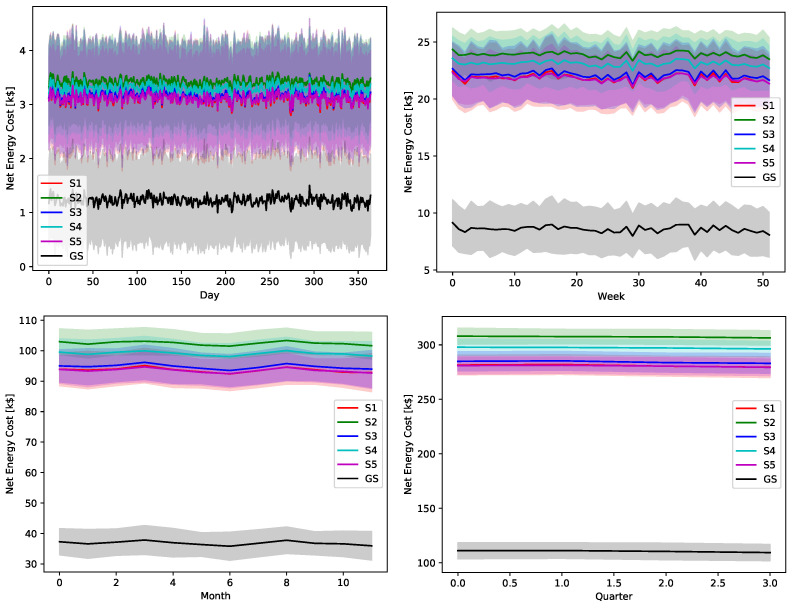
The total operation cost in terms of net energy purchase and sell revenue with a global certainty level $U_{50}$.

**Figure 21:**
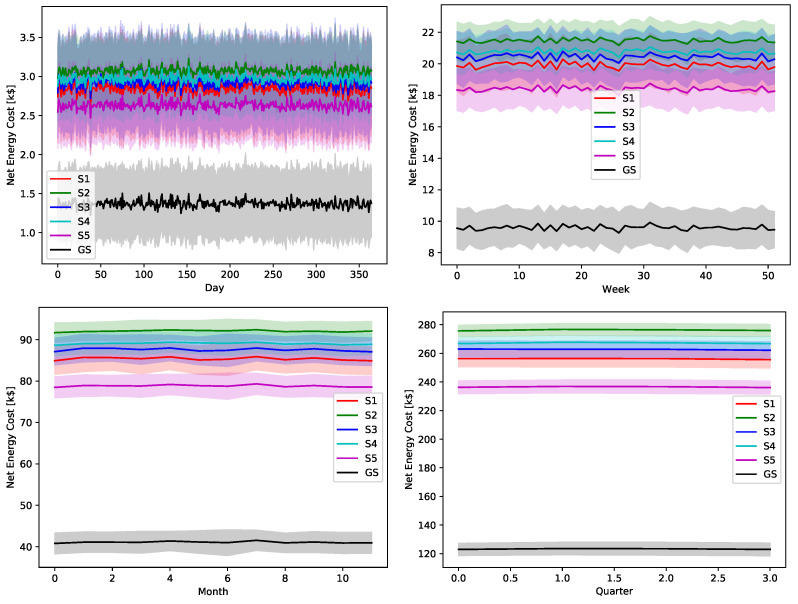
The total operation cost in terms of net energy purchase and sell revenue with a global certainty level $U_{75}$.

**Figure 22:**
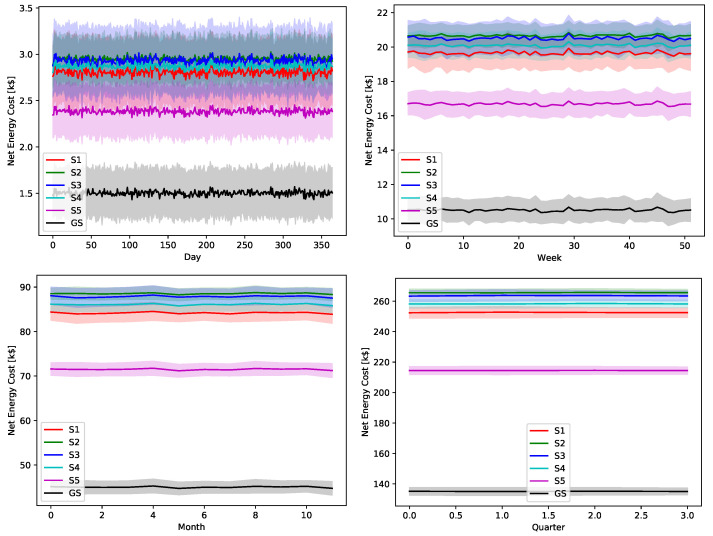
The total operation cost in terms of net energy purchase and sell revenue with a global certainty level $U_{95}$.

The ultimate impact of the proposed greedy scheduler is proved by Figure 18, Figure 19, Figure 20, Figure 21 and Figure 22. The total operational energy cost of the considered system is shown with different global uncertainty levels and different evaluation periods. The success of the proposed scheduler is clear; however, the prominent disparity between the operational costs of any scheduling policy and the proposed greedy scheduler proves its potential in achieving its ultimate goal. Although the greedy scheduler beats all the other schedulers all the time, the improvement is clearer and more stable when the evaluation period is increased and the global uncertainty level lowers.

**Figure 23:**
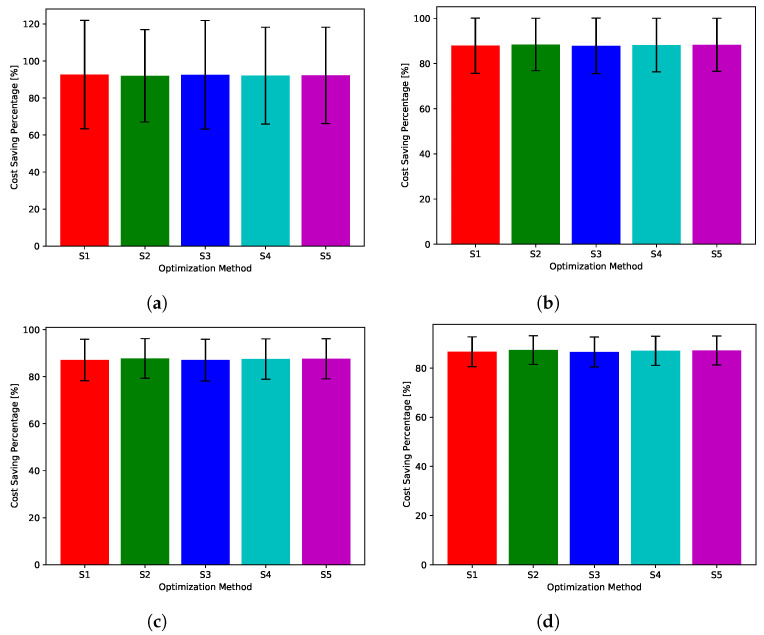
The total cost saving achieved by the proposed greedy scheduler when compared with scheduling policies fixed uncertainty level $U_{10}$. (**a**) Monthly, (**b**) quarterly, (**c**) semiannually, and (**d**) annually.

**Figure 24:**
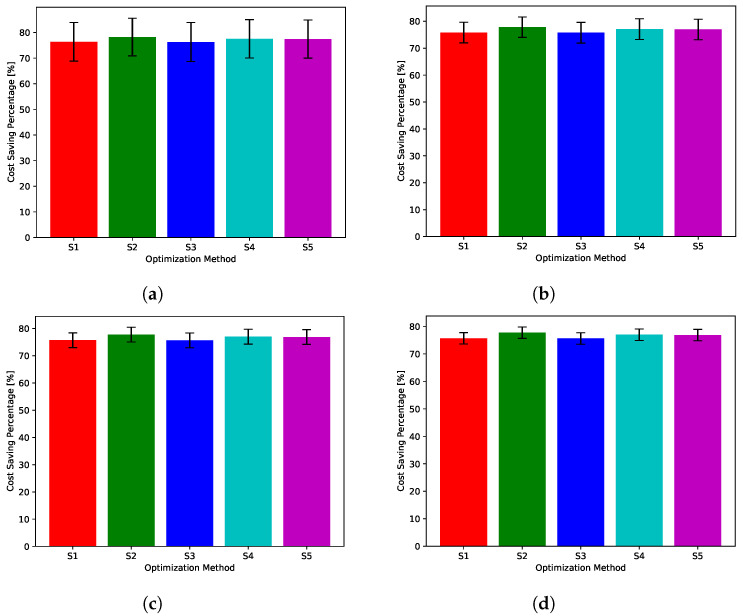
The total cost saving achieved by the proposed greedy scheduler when compared with scheduling policies fixed uncertainty level $U_{25}$. (**a**) Monthly, (**b**) quarterly, (**c**) semiannually, and (**d**) annually.

**Figure 25:**
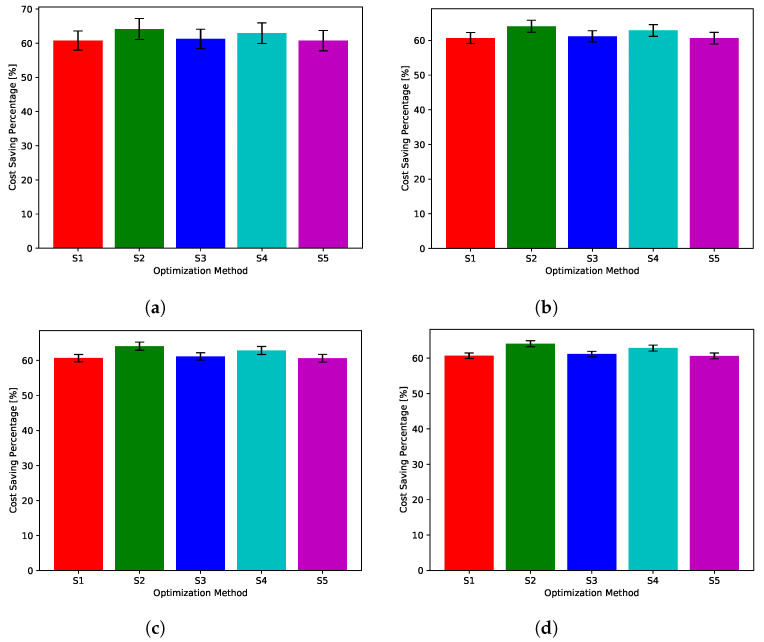
The total cost saving achieved by the proposed greedy scheduler when compared with scheduling policies fixed uncertainty level $U_{50}$. (**a**) Monthly, (**b**) quarterly, (**c**) semiannually, and (**d**) annually.

**Figure 26:**
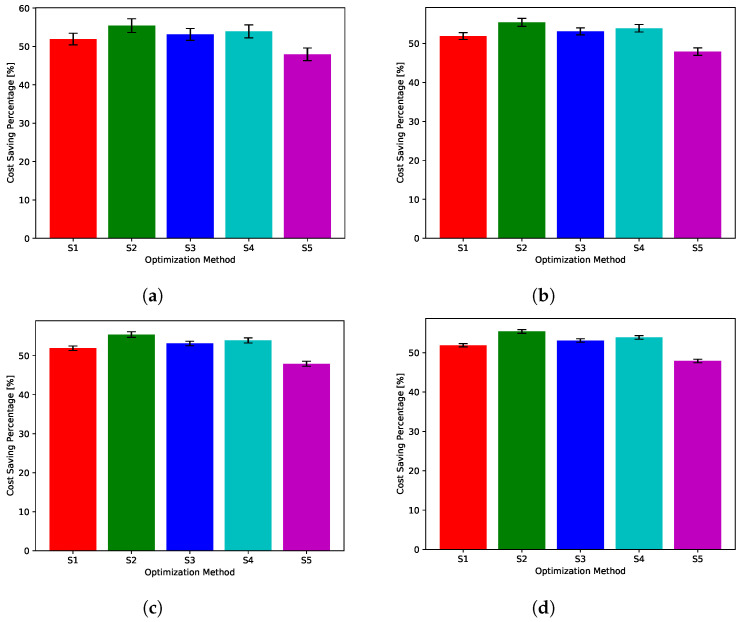
The total cost saving achieved by the proposed greedy scheduler when compared with scheduling policies fixed uncertainty level $U_{75}$. (**a**) Monthly, (**b**) quarterly, (**c**) semiannually, and (**d**) annually.

**Figure 27:**
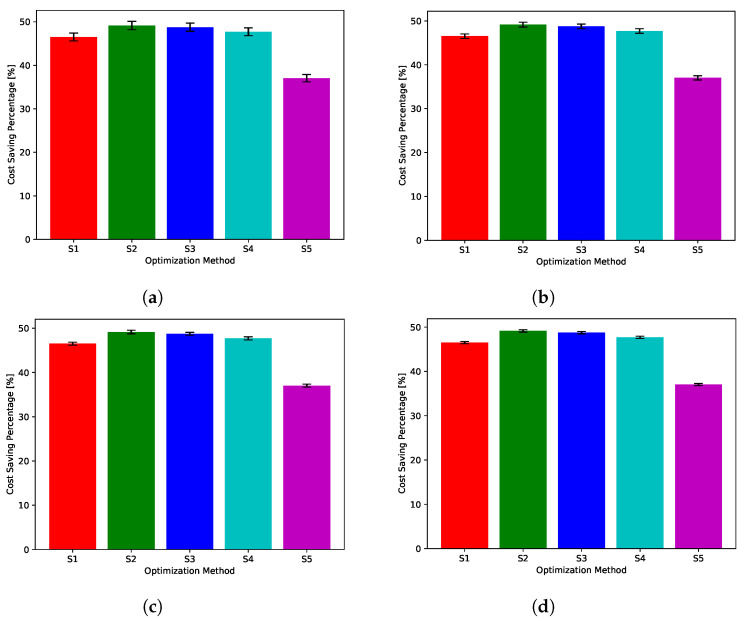
The total cost saving achieved by the proposed greedy scheduler when compared with scheduling policies fixed uncertainty level $U_{95}$. (**a**) Monthly, (**b**) quarterly, (**c**) semiannually, and (**d**) annually.

While Figure 18, Figure 19, Figure 20, Figure 21 and Figure 22 show absolute comparisons between energy costs of the considered system with and without the proposed scheduler, there is still a need for some normalized metric that can better express its effectiveness in cost saving. This is achieved by considering a normalized metric, which is calculated as the ratio between the operational costs with a single specific decision and its counterpart when applying the proposed scheduler. Figure 23, Figure 24, Figure 25, Figure 26 and Figure 27 highlight a direct measure of the savings achieved by the proposed greedy scheduler against the cases of adopting every single scheduler from *S*1–*S*5 all the time. For the highest uncertainty level, $U_{10}$, the greedy scheduler succeeded in saving about 80–85% of system’s energy cost, when calculated monthly, quarterly, semiannually, or annually. These saving values decreases a bit with the increase in the certainty values of the global uncertainty levels; however, the saving is around fifties percent in the least uncertain cases, which is still a considerably high saving achievement.

Table 6 compares the achieved results by some schedulers in the literature that delivered satisfactory performance. In each case, a basic scenario is considered from which the figures in the second column was obtained. For instance, in [1,58], the deterministic optimizers were utilized with different scenarios. The advanced techniques such as those investigated in [12,40], respectively, demonstrated better results compared to the deterministic optimizers. Despite the figures in Table 6 depending on the current prices and the nature of the considered microgrids, it is obvious that the developed heuristic greedy approach gives satisfactory results compared to the other schedulers.

## 7. Conclusions

In this work, a greedy charging/discharging scheduler is proposed for heuristic management of V2G facilities. By nature, such facilities comprise very large amounts of correlated and uncorrelated stochastic parameters. To capture such sophisticated interactions, state-of-the-art methods have to develop complex stochastic models or go into tedious training processes. The proposed approach presents a model-free simple, yet effective, method that depends on efficient instantaneous evaluation of promising candidate operational decisions energy trade. The proposed approach makes use of the batteries of the parking EVs by considering them as available energy storage with stochastic numbers and capacities. The ICTMC Monte Carlo method was used to develop a comprehensive data simulator to generate evaluation data. The performance of the proposed method was evaluated using data generated by this simulator of an effective duration of 500 operational years. The proposed scheduler succeeded in overcoming the stochastic uncertainties exhibited by the system. The evaluation results proved the effectiveness of the proposed greedy scheduler in operational cost saving ranges from 50–85% depending on different operational circumstances.

## Figures and Tables

**Figure 13:**
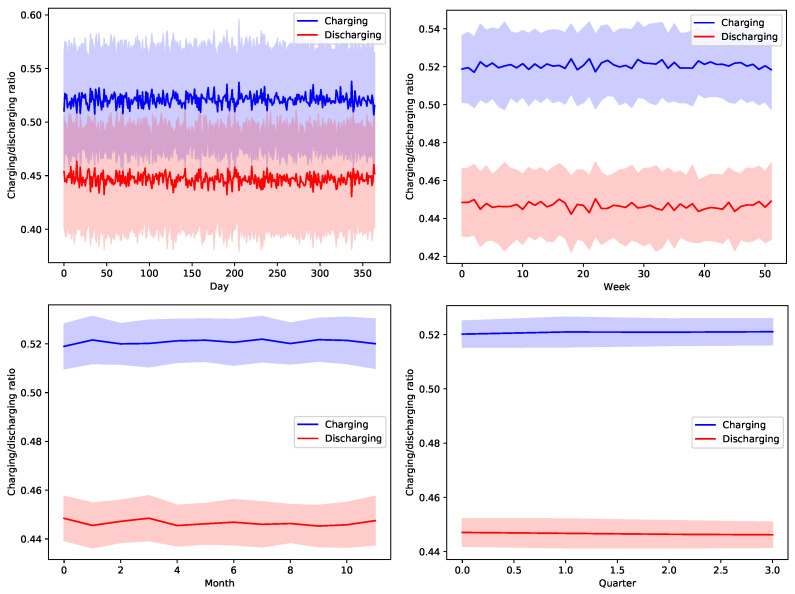
The average temporal charging and discharging ratios of EVs according to the greedy policy with a global certainty level $U_{10}$.

**Figure 14:**
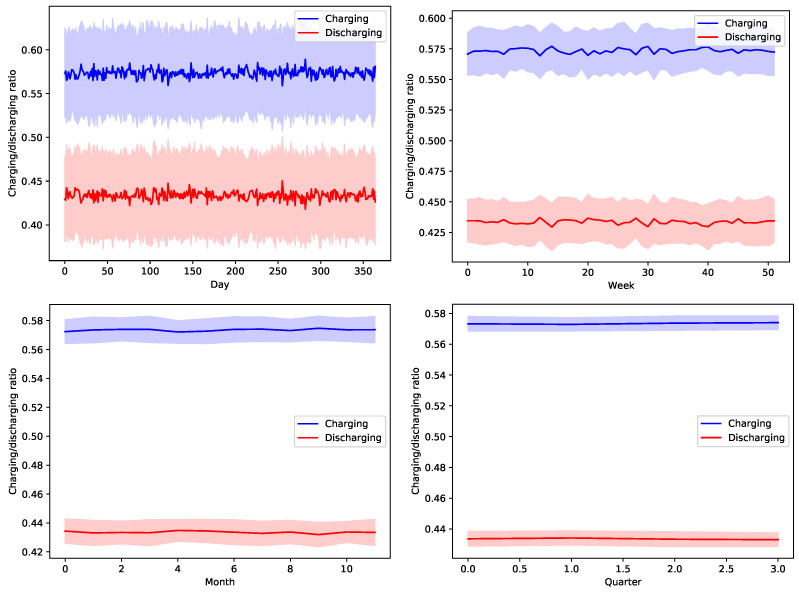
The average temporal charging and discharging ratios of EVs according to the greedy policy with a global certainty level $U_{25}$.

**Figure 15:**
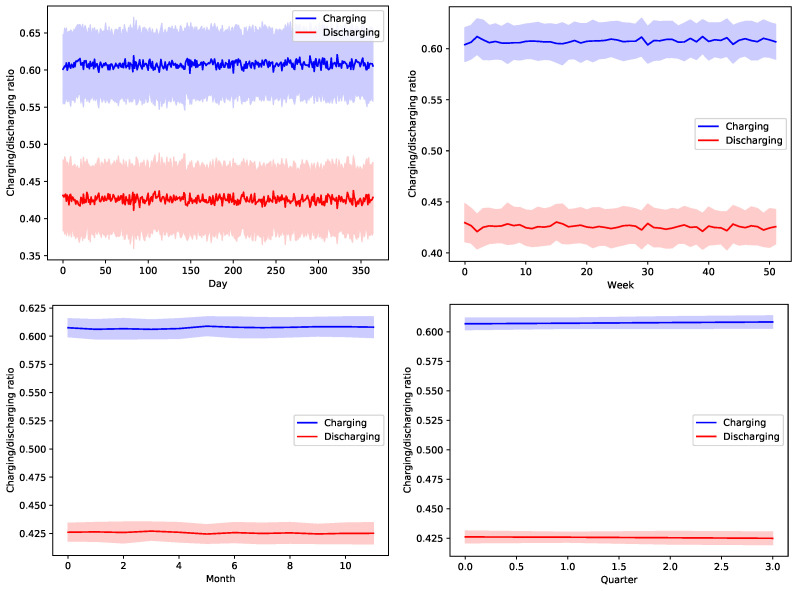
The average temporal charging and discharging ratios of EVs according to the greedy policy with a global certainty level $U_{50}$.

**Figure 16:**
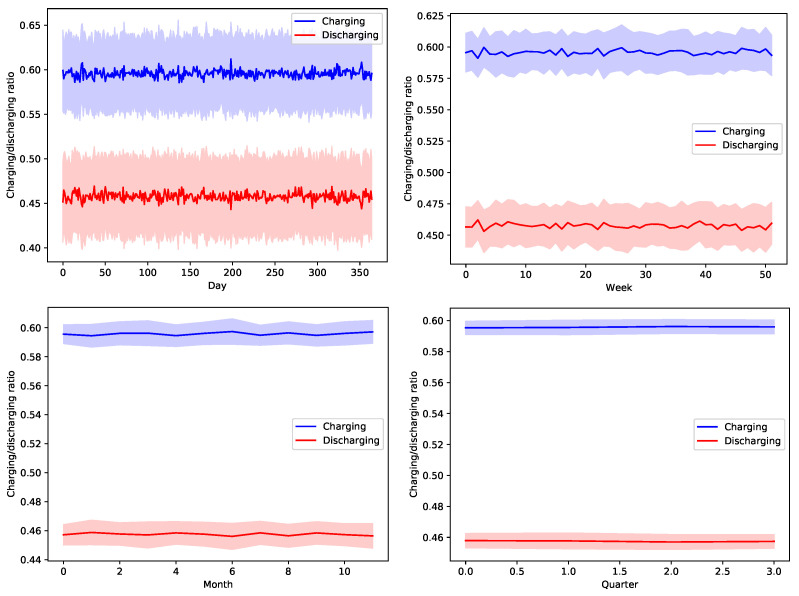
The average temporal charging and discharging ratios of EVs according to the greedy policy with a global certainty level $U_{75}$.

**Figure 17:**
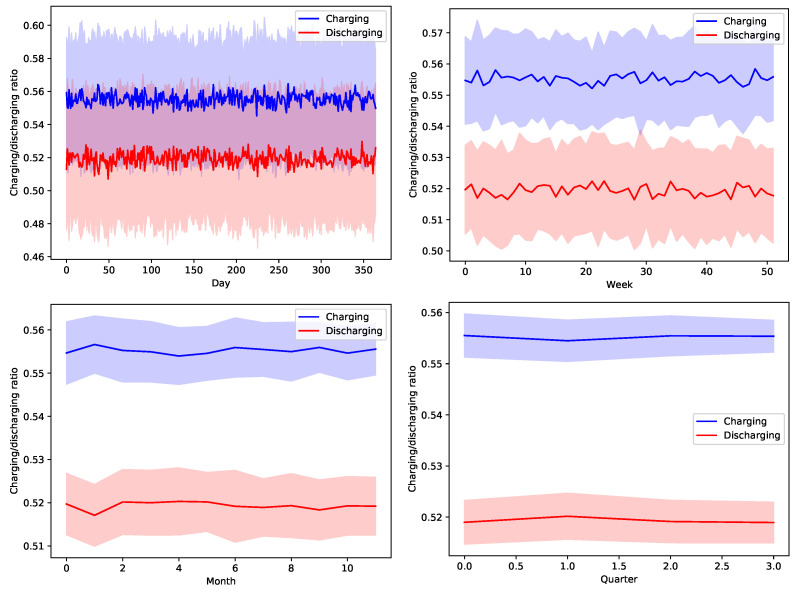
The average temporal charging and discharging ratios of EVs according to the greedy policy with a global certainty level $U_{95}$.

**Table 1:** Microgrid technical parameters.

Parameter	Value	Unit
Number of vehicles	200	Vehicle
Charger capacity	0.02	MWh
Peak load	0.96	Mwh
Converter efficiency	85	%
Battery capacity	0.04	Mwh
Battery efficiency	80	%

**Table 2:** The candidate decisions that are evaluated by the proposed greedy scheduler.

Decision	Number of Charging EVs	Number of Discharging EVs
S1	$N_{ch}$	0
S2	0	$N_{dch}$
S3	$N_{ch}$	$N_{dch\_min}$
S4	$N_{ch\_min}$	$N_{dch}$
S5	0	0

**Table 3:** Historical arrival and departure rates of EVs.

Day Quarter	Q1	Q2	Q3	Q4
Arrival Rate	70.0	60.0	15.0	10.0
Departure Rate	0.33	0.30	0.50	0.40

**Table 4:** The used global uncertainty levels in the conducted experimentation. Each certainty level implies a specific sample percentage, which is the portion of the drawn samples that swap around ± half of the mean values of the considered parameter.

Uncertainty Level	Z-Score	Samples Percentage
$U_{10}$	0.1256	10%
$U_{25}$	0.32	25%
$U_{50}$	0.6745	50%
$U_{75}$	1.15	75%
$U_{95}$	1.96	95%

**Table 5:** The average scheduling percentages of every charging/discharging decision with different global uncertainty level.

	S1	S2	S3	S4	S5
$U_{10}$	49.21±2.27	42.07±2.23	2.22±0.67	1.78±0.61	4.72±1.15
$U_{25}$	50.89±2.30	37.21±2.16	5.29±1.03	4.17±0.93	2.44±0.71
$U_{50}$	49.56±2.28	31.66±2.05	9.23±1.36	7.13±1.21	2.42±0.70
$U_{75}$	44.46±2.12	30.32±2.03	11.68±1.59	10.65±1.41	2.89±0.75
$U_{95}$	38.34±1.75	31.69±1.96	11.18±1.62	15.94±1.75	2.85±0.76

**Table 6:** Effectiveness of the previous schedulers for the V2G operation.

Technique	Best Cost Saving (%)
Markov chain [12]	24
Reinforcement learning [37]	57
Genetic algorithm [58]	5.97
Mixed-integer nonlinear programming [40]	29
First in first served [1]	3.4
Proposed stochastic greedy algorithm	85

## References

[1] Fernandez G.S., Krishnasamy V., Kuppusamy S., Ali J.S., Ali Z.M., El-Shahat A., Abdel Aleem S.H. (2020). Optimal Dynamic Scheduling of Electric Vehicles in a Parking Lot Using Particle Swarm Optimization and Shuffled Frog Leaping Algorithm. Energies.

[2] Fotopoulou M., Rakopoulos D., Blanas O. (2021). Day Ahead Optimal Dispatch Schedule in a Smart Grid Containing Distributed Energy Resources and Electric Vehicles. Sensors.

[3] CU https://www.copper.org/environment/sustainable-energy/electric-vehicles/.

[4] Khazraj H., Khanghah B.Y., Ghimire P., Martin F., Ghomi M., da Silva F.F., Bak C.L. (2019). Optimal operational scheduling and reconfiguration coordination in smart grids for extreme weather condition. IET Gener. Transm. Distrib..

[5] Abo-Elyousr F.K., Aref M., Elnozahy A. Practicality of Wave Energy Conversion Systems at the Northern Egyptian Sea Water. Proceedings of the 2021 22nd International Middle East Power Systems Conference (MEPCON).

[6] Nasir T., Raza S., Abrar M., Muqeet H.A., Jamil H., Qayyum F., Cheikhrouhou O., Alassery F., Hamam H. (2021). Optimal Scheduling of Campus Microgrid Considering the Electric Vehicle Integration in Smart Grid. Sensors.

[7] Quynh N.V., Ali Z.M., Alhaider M.M., Rezvani A., Suzuki K. (2021). Optimal energy management strategy for a renewable-based microgrid considering sizing of battery energy storage with control policies. Int. J. Energy Res..

[8] Elavarasan R.M., Shafiullah G., Raju K., Mudgal V., Arif M.T., Jamal T., Subramanian S., Balaguru V.S., Reddy K., Subramaniam U. (2020). COVID-19: Impact analysis and recommendations for power sector operation. Appl. Energy.

[9] Abo-Elyousr F.K., Guerrero J.M., Ramadan H.S. (2021). Prospective hydrogen-based microgrid systems for optimal leverage via metaheuristic approaches. Appl. Energy.

[10] Rojas-Dueñas G., Riba J.R., Moreno-Eguilaz M. (2021). CNN-LSTM-Based Prognostics of Bidirectional Converters for Electric Vehicles’ Machine. Sensors.

[11] Jawad M., Qureshi M.B., Ali S.M., Shabbir N., Khan M.U.S., Aloraini A., Nawaz R. (2020). A Cost-Effective Electric Vehicle Intelligent Charge Scheduling Method for Commercial Smart Parking Lots Using a Simplified Convex Relaxation Technique. Sensors.

[12] Mortaz E., Valenzuela J. (2017). Microgrid energy scheduling using storage from electric vehicles. Electr. Power Syst. Res..

[13] Aldosary A., Rawa M., Ali Z.M., Razmjoo A., Rezvani A. (2021). Energy management strategy based on short-term resource scheduling of a renewable energy-based microgrid in the presence of electric vehicles using *θ*-modified krill herd algorithm. Neural Comput. Appl..

[14] Thomas D., Deblecker O., Ioakimidis C.S. (2018). Optimal operation of an energy management system for a grid-connected smart building considering photovoltaics’ uncertainty and stochastic electric vehicles’ driving schedule. Appl. Energy.

[15] Koltsaklis N.E., Giannakakis M., Georgiadis M.C. (2018). Optimal energy planning and scheduling of microgrids. Chem. Eng. Res. Des..

[16] Tao H., Ahmed F.W., Latifi M., Nakamura H., Li Y. (2021). Hybrid Whale Optimization and Pattern search algorithm for Day-Ahead Operation of a Microgrid in the Presence of Electric Vehicles and Renewable Energies. J. Clean. Prod..

[17] Zhang T., Pota H., Chu C.C., Gadh R. (2018). Real-time renewable energy incentive system for electric vehicles using prioritization and cryptocurrency. Appl. Energy.

[18] Qiu D., Ye Y., Papadaskalopoulos D., Strbac G. (2020). A deep reinforcement learning method for pricing electric vehicles with discrete charging levels. IEEE Trans. Ind. Appl..

[19] Van-Hai Bui A.H., Kim H.M. (2017). A Strategy for Optimal Microgrid Operation Considering Vehicle-to-Grid Service. Int. J. Control Autom..

[20] Liao J., Liu T., Tang X., Mu X., Huang B., Cao D. (2020). Decision-making Strategy on Highway for Autonomous Vehicles using Deep Reinforcement Learning. IEEE Access.

[21] Pfeifer A., Dobravec V., Pavlinek L., Krajačić G., Duić N. (2018). Integration of renewable energy and demand response technologies in interconnected energy systems. Energy.

[22] Carpinelli G., Mottola F., Proto D., Russo A. (2017). Operation of Plug-In Electric Vehicles for Voltage Balancing in Unbalanced Microgrids. Development and Integration of Microgrids.

[23] Mortaz E., Valenzuela J. (2018). Optimizing the size of a V2G parking deck in a microgrid. Int. J. Electr. Power Energy Syst..

[24] Pfeifer A., Bošković F., Dobravec V., Matak N., Krajačić G., Duić N., Pukšec T. Building smart energy systems on Croatian islands by increasing integration of renewable energy sources and electric vehicles. Proceedings of the 2017 IEEE International Conference on Environment and Electrical Engineering and 2017 IEEE Industrial and Commercial Power Systems Europe (EEEIC/I&CPS Europe).

[25] Bracco S., Cancemi C., Causa F., Longo M., Siri S. (2018). Optimization model for the design of a smart energy infrastructure with electric mobility. IFAC-PapersOnLine.

[26] Zdunek R., Grobelny A., Witkowski J., Gnot R.I. (2021). On–Off Scheduling for Electric Vehicle Charging in Two-Links Charging Stations Using Binary Optimization Approaches. Sensors.

[27] Fathy A., Abdelaziz A.Y. (2020). Competition over resource optimization algorithm for optimal allocating and sizing parking lots in radial distribution network. J. Clean. Prod..

[28] Zakaria A., Ismail F.B., Lipu M.H., Hannan M. (2020). Uncertainty models for stochastic optimization in renewable energy applications. Renew. Energy.

[29] Zhang T.Z., Chen T.D. (2020). Smart charging management for shared autonomous electric vehicle fleets: A Puget Sound case study. Transp. Res. Part D Transp. Environ..

[30] Alipour M., Mohammadi-Ivatloo B., Moradi-Dalvand M., Zare K. (2017). Stochastic scheduling of aggregators of plug-in electric vehicles for participation in energy and ancillary service markets. Energy.

[31] Dicorato M., Forte G., Trovato M., Muñoz C.B., Coppola G. (2019). An Integrated DC Microgrid Solution for Electric Vehicle Fleet Management. IEEE Trans. Ind. Appl..

[32] Sufyan M., Rahim N., Muhammad M., Tan C., Raihan S., Bakar A. (2020). Charge coordination and battery lifecycle analysis of electric vehicles with V2G implementation. Electr. Power Syst. Res..

[33] Egbue O., Uko C. (2020). Multi-agent approach to modeling and simulation of microgrid operation with vehicle-to-grid system. Electr. J..

[34] Salehpour M.J., Tafreshi S.M. (2020). Contract-based utilization of plug-in electric vehicle batteries for day-ahead optimal operation of a smart micro-grid. J. Energy Storage.

[35] Sarparandeh M.H., Ehsan M. (2017). Pricing of Vehicle-to-Grid Services in a Microgrid by Nash Bargaining Theory. Math. Probl. Eng..

[36] Aliasghari P., Mohammadi-Ivatloo B., Alipour M., Abapour M., Zare K. (2018). Optimal scheduling of plug-in electric vehicles and renewable micro-grid in energy and reserve markets considering demand response program. J. Clean. Prod..

[37] Kim S., Lim H. (2018). Reinforcement learning based energy management algorithm for smart energy buildings. Energies.

[38] Liu Z., Wu Q., Ma K., Shahidehpour M., Xue Y., Huang S. (2018). Two-stage optimal scheduling of electric vehicle charging based on transactive control. IEEE Trans. Smart Grid.

[39] Motalleb M., Reihani E., Ghorbani R. (2016). Optimal placement and sizing of the storage supporting transmission and distribution networks. Renew. Energy.

[40] Mortaz E. (2019). Portfolio Diversification for an Intermediary Energy Storage Merchant. IEEE Trans. Sustain. Energy.

[41] Mortaz E., Vinel A., Dvorkin Y. (2019). An optimization model for siting and sizing of vehicle-to-grid facilities in a microgrid. Appl. Energy.

[42] Zhang X., Guo L., Zhang H., Guo L., Feng K., Lin J. (2019). An Energy Scheduling Strategy With Priority Within Islanded Microgrids. IEEE Access.

[43] Bracco S., Delfino F., Longo M., Siri S. (2019). Electric Vehicles and Storage Systems Integrated within a Sustainable Urban District Fed by Solar Energy. J. Adv. Transp..

[44] Fu B., Chen M., Fei Z., Wu J., Xu X., Gao Z., Wu Z., Yang Y. (2021). Research on the Stackelberg Game Method of Building Micro-grid with Electric Vehicles. J. Electr. Eng. Technol..

[45] Ferro G., Laureri F., Minciardi R., Robba M. (2019). A predictive discrete event approach for the optimal charging of electric vehicles in microgrids. Control Eng. Pract..

[46] Luis S.Y., Reina D.G., Marín S.L.T. (2021). A Multiagent Deep Reinforcement Learning Approach for Path Planning in Autonomous Surface Vehicles: The Ypacaraí Lake Patrolling Case. IEEE Access.

[47] Yanes Luis S., Gutiérrez-Reina D., Toral Marín S. (2021). A Dimensional Comparison between Evolutionary Algorithm and Deep Reinforcement Learning Methodologies for Autonomous Surface Vehicles with Water Quality Sensors. Sensors.

[48] Malek Y.N., Najib M., Bakhouya M., Essaaidi M. (2021). Multivariate deep learning approach for electric vehicle speed forecasting. Big Data Min. Anal..

[49] Lu C., Gao L., Yi J., Li X. (2020). Energy-efficient scheduling of distributed flow shop with heterogeneous factories: A real-world case from automobile industry in China. IEEE Trans. Ind. Inform..

[50] InsideEVs (2020). Top 10 Best-Selling Plug-In Electric Cars in U.S.—2019 Edition. https://insideevs.com/news/392375/top-10-electric-cars-sales-us-2019/.

[51] Wu X., Wang X., Qu C. (2014). A hierarchical framework for generation scheduling of microgrids. IEEE Trans. Power Deliv..

[52] Zidan A., El-Saadany E. (2015). Incorporating customers’ reliability requirements and interruption characteristics in service restoration plans for distribution systems. Energy.

[53] Gabbar H.A., Zidan A. (2016). Optimal scheduling of interconnected micro energy grids with multiple fuel options. Sustain. Energy Grids Netw..

[54] Li Y.F., Lin Y.H., Zio E. Stochastic Modeling by Inhomogeneous Continuous Time Markov Chains. Proceedings of the EURO Workshop on Stochastic Modelling.

[55] Iannuzzi D., Franzese P. (2021). Ultrafast charging station for electrical vehicles: Dynamic modelling, design and control strategy. Math. Comput. Simul..

[56] Lipowski A., Lipowska D. (2012). Roulette-wheel selection via stochastic acceptance. Phys. A Stat. Mech. Appl..

[57] PJM https://www.pjm.com/.

[58] Moradijoz M., Moghaddam M.P., Haghifam M., Alishahi E. (2013). A multi-objective optimization problem for allocating parking lots in a distribution network. Int. J. Electr. Power Energy Syst..

